# MARCHF8-mediated ubiquitination via TGFBI regulates NF-κB dependent inflammatory responses and ECM degradation in intervertebral disc degeneration

**DOI:** 10.1371/journal.pone.0314021

**Published:** 2025-01-03

**Authors:** Xingpeng Zhang, Guang Li, Fang Tan, Tao Yu, Chengping Xu, Kai Li, Feng Zhang, Meiyan Zhang, Jian Wang

**Affiliations:** 1 Department of Orthopedics, Shanghai Pudong New Area People’s Hospital, Shanghai, China; 2 Department of Traumatic Surgery, Emergency Center, Shanghai East Hospital, Tongji University School of Medicine, Shanghai, China; 3 Shanghai Circle Harmony Xinyong Clinic, Shanghai, China; University of Crete, GREECE

## Abstract

**Aim:**

To explore the role of the hub gene Transforming Growth Factor Beta Induced (TGFBI) in Intervertebral disc degeneration (IDD) pathogenesis and its regulatory relationship with Membrane Associated Ring-CH-Type Finger 8 (MARCHF8).

**Background:**

IDD is a prevalent musculoskeletal disorder leading to spinal pathology. Despite its ubiquity and impact, effective therapeutic strategies remain to be explored.

**Objective:**

Identify key modules associated with IDD and understand the impact of TGFBI on nucleus pulposus (NP) cell behavior, extracellular matrix (ECM)-related proteins, and the Nuclear Factor kappa-light-chain-enhancer of Activated B cells (NF-κB) signaling pathway.

**Methods:**

The GSE146904 dataset underwent Weighted Gene Co-Expression Network Analysis (WGCNA) for key module identification and Differentially Expressed Genes (DEGs) screening. Intersection analysis, network analysis, and co-expression identified TGFBI as a hub gene. *In vitro* experiments delved into the interplay between TGFBI and MARCHF8 and their effects on NP cells.

**Results:**

WGCNA linked the MEturquoise module with IDD samples, revealing 145 shared genes among DEGs. *In vitro* findings indicated that MARCHF8 determines TGFBI expression. TGFBI boosts apoptosis and ECM breakdown in Lipopolysaccharide-stimulated (LPS-stimulated) NP cells. Altering TGFBI levels modulated these effects and the NF-κB signaling pathway, influencing inflammatory cytokine concentrations. Moreover, MARCHF8 ubiquitination controlled TGFBI expression.

**Conclusion:**

TGFBI, modulated by MARCHF8, significantly influences IDD progression by affecting NP cell apoptosis, ECM degradation, and inflammation through the NF-κB signaling pathway.

## Introduction

Intervertebral disc degeneration (IDD) is a widespread issue that affects the intervertebral discs (IVDs) in our spine. These IVDs are the soft, cushion-like structures between the vertebrae that provide flexibility and support to the spine [1,2]. It leads to less water and more fibrosis in the discs, which reduces their stability and can cause spinal issues [3,4]. The progression of IDD is attributed to a combination of intrinsic and extrinsic factors, including genetic predisposition, age-related changes, mechanical stress, inflammation, and oxidative stress [5,6]. Together, these factors cause the IVD to progressively worsen and eventually lead to its regression [7]. IDD poses a considerable burden to global health, and its prevalence increases as the population ages [8,9]. Although IDD-related mortality is relatively low, its impact on quality of life is substantial, often resulting in chronic pain, reduced mobility, and reduced productivity [10–12]. The current field of IDD research covers a wide range of research avenues, ranging from molecular mechanisms of degeneration to clinical intervention and management strategies [13,14]. Treatment options vary from conservative approaches such as physical therapy to surgical interventions such as spinal fusion or artificial disc replacement [13,15]. Prognostic indicators, including imaging biomarkers and molecular signatures, are being explored to predict disease progression and treatment outcomes. New diagnostic markers, therapeutic methods, and prognosis indicators must be created immediately given the huge socioeconomic burden of IDD [16].

The current understanding of the molecular mechanisms of IDD is one of the key focuses of research [17]. The development of IDD involves a variety of molecular mechanisms, including apoptosis, inflammatory responses, matrix degradation, and imbalances in extracellular matrix synthesis [18]. It is still not fully understood how these mechanisms interact and their exact roles in the pathologic process. Studies have shown that gene expression regulation plays an important role in the development and progression of IDD [19]. However, the regulatory networks of specific genes and their effects on disc cell function still require further investigation. Inflammatory pathways and immune responses play a key role in the development of IDD [20,21]. Understanding how these pathways change in different patient populations and their impact on disease progression is an important direction for current research.

Transforming Growth Factor Beta Induced (TGFBI) is an extracellular matrix protein. It plays a role in cell adhesion, migration, and maintaining tissue health [22–24]. Its importance extends beyond its structure and function to encompass regulatory aspects of various diseases. Studies have shown that aberrant expression of TGFBI is associated with the development of a variety of cancers and may play a tumor-promoting or tumor-suppressing role in the tumor microenvironment [25]. The multifaceted contribution of TGFBI in a range of pathologies ranging from cancer to degenerative diseases is evident. Multiple studies have revealed its involvement in tumor progression, metastasis, and drug resistance, emphasizing its complex relationship with malignancy [26]. For example, in cholangiocarcinoma, the Lin-28 homolog B (LIN28B)/ Transforming Growth Factor Beta (TGF-β)/TGFBI feedback loop encourages cell migration and carcinogenesis [27]. Furthermore, its influence extends to ocular diseases such as corneal dystrophies, emphasizing its multiple effects on tissue integrity and function [28,29]. Notably, the effect of TGFBI on cancer stemness and its modulation of immune responses in the tumor microenvironment underscores its contribution to tumorigenesis, and TGFBI can activate the Focal Adhesion Kinase-Mitogen-Activated Protein Kinase—Extracellular signal-Regulated Kinase (FAK-MAPK-ERK) signaling pathway to alter the tumor microenvironment [17,30]. The extracellular matrix (ECM) is a network of proteins and carbohydrates found outside cells. It provides structural support to tissues and plays a role in cell communication and signaling. In degenerative diseases, the interaction of TGFBI with extracellular matrix components and its role in cellular senescence highlight its involvement in disease progression and tissue degeneration [31]. Recent studies have shown that TGFBI may affect the degradation of the extracellular matrix in the intervertebral disc, thereby contributing to the disc degeneration cascade. Given its complex involvement in cellular and matrix dynamics, deciphering the exact mechanism underlying the link between TGFBI and IDD could provide valuable insights into disease mechanisms and potential new avenues of intervention.

Membrane Associated Ring-CH-Type Finger 8 (MARCHF8) protein is an E3 ubiquitin ligase that plays a significant role within cells. This protein belongs to the Membrane Associated Ring Finger (MARCH) family, which adds ubiquitin molecules to target proteins, marking them for degradation or regulating their functions [32]. MARCHF8 is crucial in modulating immune responses, cellular signal transduction, and protein homeostasis [33]. Whether there is an interactive relationship between MARCHF8 and TGFBI, and whether it plays a role in IDD, remains to be investigated. MARCHF8, as a positive regulator of IFN-I signaling, is involved in the regulation of antiviral natural immune signaling pathways [34]. During viral infection, MARCHF8 influences viral replication and host immune response by affecting the autophagy process and IFN-I signaling pathway [34]. This suggests that MARCHF8 plays an important role in antiviral natural immunity and provides a new perspective for a deeper understanding of the molecular mechanisms of host-pathogen interactions.

IDD is a complex condition that involves the deterioration of the intervertebral discs, which are crucial for spinal flexibility and support. TGFBI, an extracellular matrix protein, has been implicated in IDD due to its role in maintaining tissue integrity and homeostasis. MARCHF8 is an E3 ubiquitin ligase that may have a functional relationship with TGFBI. The study of TGFBI and MARCHF8 within the context of IDD is crucial as it addresses a significant knowledge gap in the field. TGFBI’s role in extracellular matrix dynamics and MARCHF8’s influence on protein ubiquitination represent key pathways that, once better understood, could lead to the discovery of new therapeutic targets for IDD. Our research has the potential to not only enhance our comprehension of the disease’s molecular underpinnings but also to inform the development of targeted treatments and diagnostic tools. The implications of this work could be transformative for future IDD management strategies, offering patients more effective options for treatment and potentially slowing disease progression.

In this study, we identified the key gene TGFBI related to IDD through bioinformatics analysis and studied the effects of TGFBI on nucleus pulposus (NP) cell inflammatory response, ECM degradation, and NF-κB pathway in IDD by *in vitro* cell experiments. MARCHF8-mediated ubiquitination regulates TGFBI expression and NF-κB signaling pathway-dependent regulation of NP cell inflammatory response and ECM degradation. These insights highlight the MARCHF8-TGFBI axis as a key regulator of NF-κB-driven inflammation and ECM degradation in NP cells. This suggests that managing IDD by targeting this axis has therapeutic potential.

## Material and methods

### Acquiring and analyzing the GSE146904 dataset from the Gene Expression Omnibus (GEO) database

From the GEO database (http://www.ncbi.nlm.nih.gov/geo), we retrieved the GSE146904 dataset. Among them, 10 cases of soft NP samples of lumbar intervertebral disc, including 5 cases each of disc herniation, prolapse (DH), and disc degeneration (DS). Then, the differentially expressed genes (DEGs) of the two sets of samples were examined and filtered by the GEO2R program and the fold change (FC) >2 was up-regulated, <0.5 was down-regulated, all *p*<0.05. This ensures that the identified gene expression differences have biological significance and may play a crucial role in the disease process. The threshold is commonly utilized as it strikes a balance between sensitivity and specificity, aiding in the filtration of genes with minor expression changes that may not be biologically relevant, while still capturing genes with substantial alterations.

### Weighted gene co-expression network analysis (WGCNA) analysis of the GSE146904 dataset

The "WGCNA" package of R software constructed a gene co-expression network for all genes in the GSE146904 dataset and determined the optimal soft threshold to make the gene network conform to the scale-free topology. Next, feature heatmaps for the 10 samples were generated based on the gene network. Then, according to the difference of gene expression level, it is divided into different gene modules. Finally, the association of the modules with two sample groups in the GSE146904 dataset is evaluated. Modules exhibiting the highest correlation coefficients were considered critical modules. The 1632 DEGs in the GSE146904 dataset and the 1645 genes in the turquoise module were visually analyzed using the Venn diagram package of the R language.

### Integrative network analysis reveals hub genes in IDD

We used the Interacting Gene Retrieval Search Tool (Search Tool for the Retrieval of Interacting Genes/Proteins, STRING) to create a Protein-Protein Interaction Network (PPI) network among 145 overlapping genes. The PPI network was then imported into Cytoscape for target clustering analysis and core target screening by Molecular Complex Detection (MCODE). Combined with the DMNC algorithm of the CytoHubba plug-in in Cytoscape, the hub genes were analyzed. The 19 genes screened by MCODE and the 20 genes screened by DMNC were analyzed by Venn diagram. Employing datasets GSE146904 and GSE34095 sourced from the GEO database, encompassing 3 representative samples each of degenerative disc cases and non-degenerative disc controls, we conducted an in-depth analysis of the expression profiles of 14 specific genes across the case and control sample cohorts.

### Cell culture and transfection

ScienCell Research Laboratories (Carlsbad, CA, USA) provided the human NP cells, which were grown in Dulbecco’s modified eagle medium (DMEM, Gibco, Waltham, MA, USA) with 10% fetal bovine serum (FBS; Gibco, Waltham, MA, USA), 100 U/ml penicillin, and 100 μg/ml streptomycin (Gibco, Waltham, MA, USA). 37°C and 5% CO_2_ were used to keep the cells alive. In order to mimic the inflammatory state of intervertebral disc disease, the cells were treated with LPS (Sigma, St. Louis, MO, USA), and a BAY 11–7082 inhibitor (Sigma, St. Louis, MO, USA) was used to examine the impact of proteins associated to the NF-κB signaling pathway. Lipofectamine 2000 (Invitrogen, San Diego, CA, USA) was used for the transfection of NP cells in accordance with the manufacturer’s instructions, siRNAs were transfected with si-TGFBI (si-TGFBI-1: 5’- GCGCUUGAGAUCUUCAAACAA-3’, si-TGFBI-2: 5’- CCUCACCUCUAUGUACCAGAA-3’), si-MARCHF8: 5’- GACUGGACAGGUGACUAUUUA-3’, Negative control (NC): 5’-UUCUCCGAACGUGUCACGUTT-3’ and overexpression plasmids were transfected with over-TGFBI. Collect the cells for analysis 24 hours post-transfection.

### Quantitative real-time PCR (qRT-PCR)

TRIzol reagent (Beyotime Biotechnology) should be used to extract total RNA from NP cells in accordance with the manufacturer’s instructions. The SuperScript cDNA Synthesis Kit was used to create cDNA. SYBR Green Master Mix (Applied Biosystems, USA) was used to run qRT-PCR on an AB Fast 7500 real-time instrument. The 2^^-ΔΔCt^ method was used to calculate relative gene expression.

MARCHF8 forward primers: 5′-TTCTATCACGCCATCCAGC-3′, reverse primers: 5′-GCGTGATAGAAGTGCGAGA-3′;

TGFBI forward primers: 5′-GTGTGTGCTGTGCAGAAGGT-3′, reverse primers: 5′-CATATCCAGGACAGCACTC-3′;

GAPDH forward primers: 5′-ATCATCCCTGCCTCTACTGG-3′, reverse primers: 5′-GTCAGGTCCACCACTGACAC-3′.

### Western Blotting (WB)

Protease and phosphatase inhibitors from Roche were added to RIPA lysis buffer (from Beyotime Biotechnology) to obtain protein extracts from NP cells. A BCA Protein Assay Kit from Thermo Fisher Scientific was used to measure protein concentrations. Equal amounts of proteins were divided by SDS-PAGE, put on PVDF membranes (Millipore). After transfer, the membranes were blocked with 5% non-fat milk in Tris-buffered saline containing 0.1% Tween-20 (TBST) for 1 hour at room temperature to reduce non-specific binding. The blocked membranes were incubated overnight at 4°C with primary antibodies against TGFBI, Bax, Bcl-2, Cleaved caspase-3, Cleaved caspase-9, IKK, IKK, IKK, and p-IKB (Cell Signaling Technology) (1:1000, Abcam, USA) and GAPDH (1:5000, Abcam, USA). Protein bands were detected using an enhanced chemiluminescence system (Thermo Fisher Scientific) following incubation with horseradish peroxidase (HRP)-conjugated secondary antibody (1:5000, Abcam, USA). As a loading control, GAPDH was utilized. The intensity of the bands was quantified using image analysis software and normalized to GAPDH to account for any variability in sample loading or transfer efficiency.

### Co-Immunoprecipitation (Co-IP) assay

Co-IP was conducted with a Pierce Co-Immunoprecipitation Kit from Thermo Fisher Scientific, following the provided manufacturer’s guidelines. Briefly, protein lysates were incubated with primary antibodies against TGFBI or MARCHF8 for an overnight period at 4°C with rotation. Immune complexes were subsequently washed and eluted for further WB analysis after being added to Protein A/G agarose beads.

### Flow cytometry

An Annexin V-FITC/PI Apoptosis Detection Kit (BD Biosciences) was used in accordance with the manufacturer’s instructions to identify cell apoptosis. Propidium iodide (PI) and Annexin V-FITC were used to stain NP cells, and a flow cytometer (BD FACSCanto II) was used to evaluate the apoptotic cells.

### Enzyme-Linked Immunosorbent Assay (ELISA)

Human TNF-alpha ELISA Kit, Human IL-6 ELISA Kit, and Human IL-1 beta ELISA Kit (all from Abcam) were used in accordance with the manufacturer’s instructions to measure the levels of TNF-α, IL-6, and IL-1β.

### *In vitro* ubiquitination assay

MG132 (10 μM; Selleck Chemicals) was applied to NP cells for 12 or 18 hours. Following the collection and immunoprecipitation of cell lysates with anti-PC Ab (1:50 dilution), western blotting with anti-ubiquitin Ab (1:3000; Cell Signaling Technology (CST)) was used to evaluate the results.

### Statistical analysis

Software called GraphPad Prism was used to examine the data. Post hoc tests were used after the student’s t-test or one-way ANOVA to evaluate the statistical significance. A mean ± standard deviation (SD) was used to present the results. The choice of these tests was driven by the need to compare means between groups and to assess the impact of treatments on continuous outcomes. Additionally, GraphPad Prism software was utilized for all statistical calculations, ensuring the accuracy and reliability of our results. This comprehensive approach allows for a robust analysis of our data, providing clear and defensible conclusions regarding the effects of the interventions under study. Statistics were considered significant for *p*-values <0.05.

## Results

### WGCNA analysis based on the GSE146904 dataset

**Fig 1:**
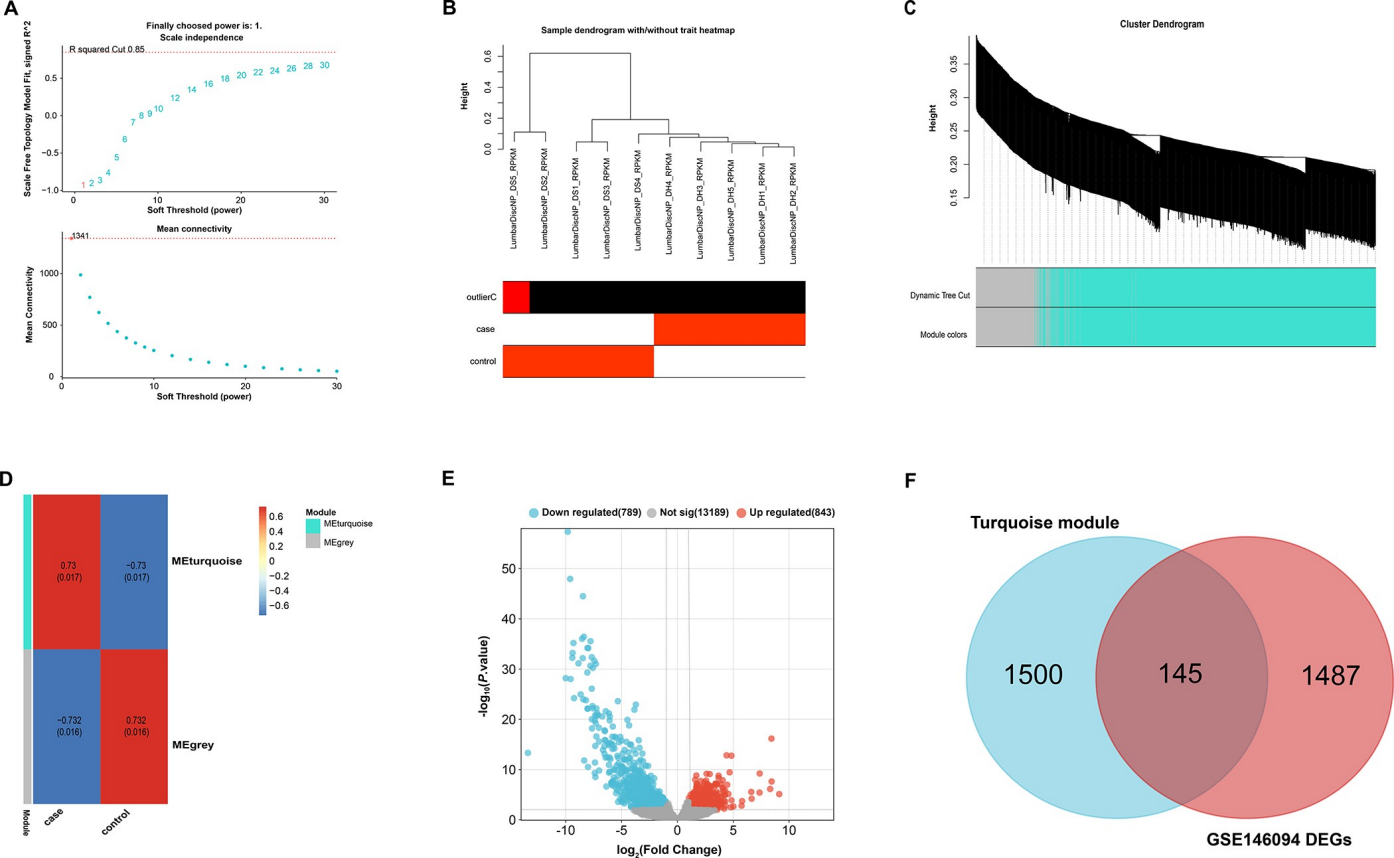
WGCNA analysis and DGEs analysis of the GSE146904 dataset. (A) Scale-free topology model fits exponential analysis and average connectivity analysis with soft-thresholding power. (B) Dendrogram of hierarchical clustering of samples, with each branch representing an individual sample. (C) Module screening is based on the clustering of gene expression patterns, with a clustered dendrogram of genes at the top and color-coded modules at the bottom representing groups of genes with similar expression patterns. (D) The heatmap of the correlation between gene modules and clinical samples. green and gray represent different modules, and red and blue represent the correlation between modules and samples. (E) Volcano map of DEGs in the GSE146904 dataset. Upregulated DEGs in IDD are highlighted in red, downregulated DEGs in blue, and insignificant genes in gray. (F) Venn diagram depicting overlapping genes of turquoise module and GSE146904 DEGs.

WGCNA is a systems biology method used to describe the patterns of co-expression of genes across conditions or samples. We created a gene co-expression network for the GSE146904 dataset using the "WGCNA" package in R software. The optimal soft threshold for this analysis was determined to be 1 (Fig 1A). Next, we generated feature heatmaps for the 10 samples in the GSE146904 dataset (Fig 1B). Genes are grouped into different modules according to their expression changes, and each module is represented by a specific color. A cluster dendrogram and feature gene adjacency heatmap were constructed (Fig 1C). The correlation between gene modules and their respective case and control groups was subsequently explored. Notably, MEturquoise was strongly positively correlated with case samples (correlation coefficient of 0.73) and equally strongly negatively correlated with control samples (correlation coefficient of -0.73) (Fig 1D). Using the GEO2R tool, we identified 789 down-regulated DEGs and 843 up-regulated DEGs from 10 samples in the GSE146904 dataset, as shown in Fig 1E. Subsequently, from the 1645 genes in these DEGs and MEturquoise, 145 common genes were selected (Fig 1F).

### Comprehensive analysis of candidate genes for intervertebral disc degeneration

**Fig 2:**
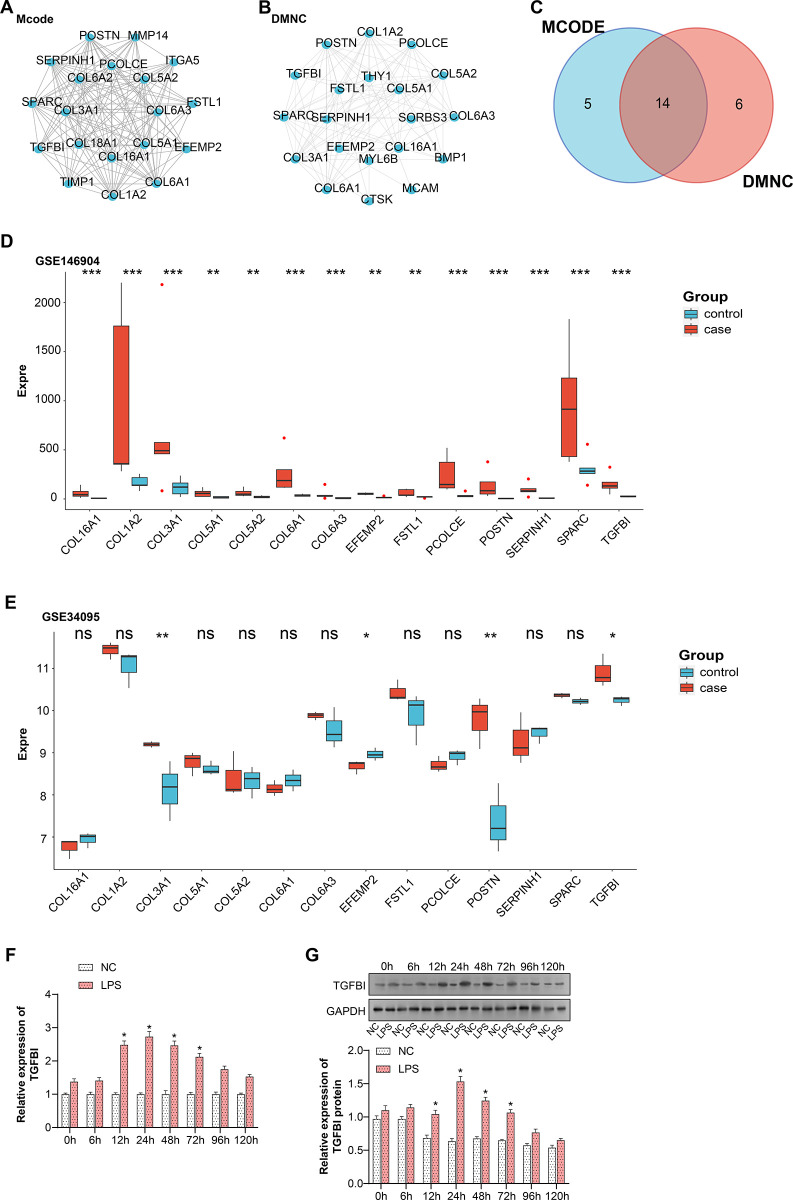
Comprehensive analysis of network clustering algorithms, gene expression, and TGFBI response in NP cells. (A) The MCODE algorithm is used to systematically identify highly interconnected gene clusters within complex biological networks. (B) Dynamic modularity of the DMNC algorithm. (C) Venn diagram depicting the intersection of gene sets identified by two different methods (MCODE and DMNC). (D) Expression levels of 14 genes in case and control samples in the GSE146904 dataset. (E) Expression levels of 14 genes in case and control samples in the GSE34095 dataset. (F) qRT-PCR was performed to detect the expression levels of TGFBI in NC cells without LPS treatment (NC group) and LPS-treated NP cells (LPS group) at 0, 6, 12, 24, 48, 72, 96 or 120 hours. (G) WB detection of protein expression levels of TGFBI in NC cells without LPS treatment (NC group) and NP cells treated with LPS (50 ng/mL) in vitro (LPS group) at 0, 6, 12, 24, 48, 72, 96 or 120 hours. *P<0.05, **P<0.01, ***P<0.001.

The MCODE Cytoscape plugin was run to find subclusters of 145 genes and identified 19 candidate genes (Fig 2A). Twenty hub genes were screened out using the DMNC algorithm of the CytoHubba plug-in in Cytoscape (Fig 2B). The Venn diagram of these two groups of genes yielded 14 overlapping genes (Fig 2C). Based on the GEO data, the expression of 14 genes in GSE146904 and GSE34094 was investigated, and it was found that in the GSE146904 dataset, the genes COL1A2, COL5A1, COL5A2, COL6A3, EFEMP2, FSTL1, SERPINH1, and TGFBI were expressed in both the case and control groups at levels were low in both the case and control groups. In the GSE34094 dataset, COL1A2 and TGFBI were highly expressed in both the case and control groups (Fig 2D–2E). The relevant p-values can be found in S1 Table. Subsequently, we investigated the temporal dynamics of TGFBI expression in NP cells after LPS stimulation. LPS is a component of the outer membrane of gram-negative bacteria, often used to mimic inflammatory responses in cell culture models. The relative expression levels of TGFBI in the NC and LPS groups, as detected by qRT-PCR, are shown in Fig 2F. Compared with the NC group, the expression level of TGFBI in the LPS-treated group showed an increasing trend from 0–24 h and a decreasing trend from 24–120 h. The WB results were highly consistent with the qPCR assay results (Fig 2G). These data suggest that LPS treatment induces TGFBI expression and that this up-regulation is evident at 0–24 h, followed by a decreasing trend at 24–120 h. The results were highly consistent with the qPCR assay results (Fig 2G). It is indicated that the 24-hour time point is critical for assessing the expression of LPS-induced inflammatory cytokines. Therefore, we chose to collect samples for analysis 24 hours after LPS treatment.

### TGFBI regulation affects NP cell expression and apoptosis

**Fig 3:**
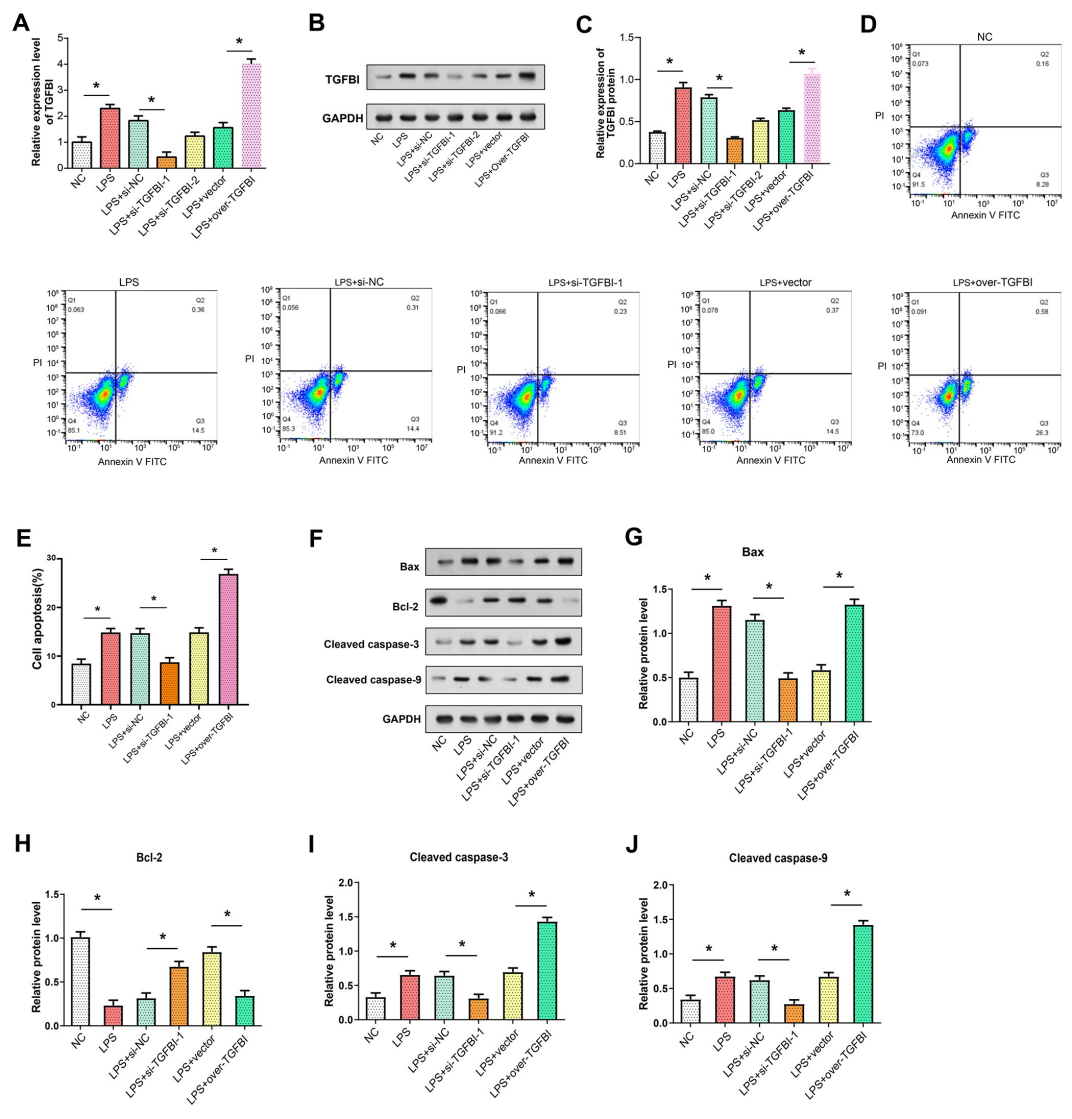
Evaluation of regulation of NP cell expression and apoptosis by TGFBI under LPS stimulation. (A) The efficiency of TGFBI in NP cells after LPS treatment was evaluated by qRT-PCR. NC (normal control), LPS (lipopolysaccharide treatment), LPS+si-NC (lipopolysaccharide with non-specific siRNA), LPS+si-TGFBI-1 (lipopolysaccharide with TGFBI-specific siRNA-1), LPS+si-TGFBI-2 (lipopolysaccharide with TGFBI-specific siRNA-2), LPS+vector (lipopolysaccharide with empty vector), LPS+over-TGFBI (lipopolysaccharide with TGFBI overexpression vector). (B) TGFBI protein levels were analyzed by WB under the same treatment conditions as the qPCR experiments, with GAPDH as an internal reference. (C) Quantitative analysis of TGFB1 protein expression relative to GAPDH, shown as the relative expression of TGFB1 protein for each treatment condition. (D) Flow cytometry assessment of TGFBI knockdown/overexpression in NP cells after treatment with LPS. (E) Histogram depicting apoptosis of TGFBI knockdown/overexpression in NP cells after LPS treatment. (F) Protein expression levels of apoptosis-related proteins (Bax, Bcl-2, Bcl-2, Cleaved caspase-3, Cleaved caspase-9) after TGFBI knockdown/overexpression in NP cells treated with LPS evaluated by WB. (G) Quantitative analysis of TGFB1 protein expression relative to GAPDH, shown as the relative expression of TGFB1 protein for each treatment condition. (F-J) Histogram depicting protein expression levels of apoptosis-related proteins (Bax, Bcl-2, Cleaved caspase-3, Cleaved caspase-9) after TGFBI knockdown/overexpression in LPS-treated NP cells. *P<0.05.

We investigated the impact of TGFBI on nucleus pulposus (NP) cells following LPS-induced treatment. By using qRT-PCR and WB to assess the expression level of TGFBI under various experimental settings following LPS treatment, the results revealed that the level of TGFBI was greater following the addition of LPS than it was under NC. Knockdown of TGFBI (si-TGFBI-1, si-TGFBI-2), the efficiency of knockdown of TGFBI-1 is higher. Compared to the LPS group, the level of TGFBI significantly increased upon overexpression of TGFBI (Fig 3A–3C). Flow cytometry analysis revealed that LPS addition increased NP cell apoptosis. Knocking down TGFBI mitigated the pro-apoptotic impact of LPS, while overexpressing TGFBI enhanced this pro-apoptotic effect (Fig 3D–3E). WB results indicated that the protein levels of Bax, Bcl-2, cleaved Caspase-3, and cleaved Caspase-9 were influenced by the knockdown and overexpression of TGFBI following LPS treatment. The findings demonstrated that LPS addition elevated the expression of apoptotic proteins such as Bax, Cleaved caspase-3, and Cleaved caspase-9. Knocking down TGFBI counteracted this effect while overexpressing TGFBI amplified it. For Bcl-2 (antiapoptotic protein), however, the results were reversed (Fig 3F–3J). This suggests that TGFBI promotes LPS-induced apoptosis in NP cells.

### Modulation of TGFBI alters ECM degradation and inflammatory responses to LPS stimulation

**Fig 4:**
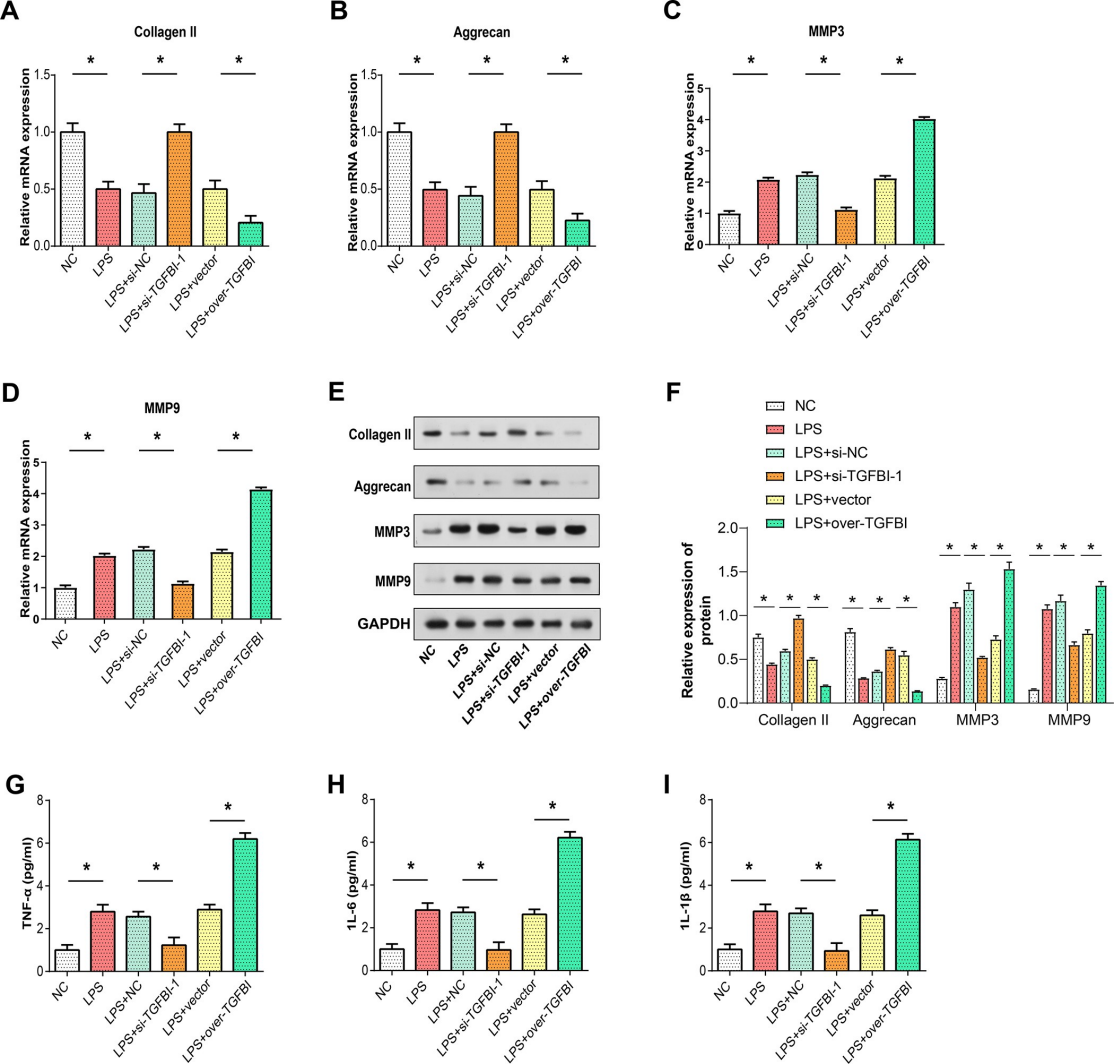
Assessment of ECM degradation markers and inflammatory factors in TGFBI knockdown/overexpression upon LPS stimulation. (A-D) qRT-PCR analysis illustrating the expression levels of ECM degradation markers (Collagen II, Aggrecan, MMP3, MMP9) following LPS treatment in TGFBI knockdown/overexpression conditions. (E) WB analysis depicting protein expression levels of ECM degradation markers (Collagen II, Aggrecan, MMP3, MMP9) upon LPS stimulation in TGFBI knockdown/overexpression conditions. (F) Quantitative analysis of Collagen, Aggrecan, MMP3, MMP9 protein expression relative to GAPDH, shown as the relative expression of Collagen, Aggrecan, MMP3, MMP9 protein for each treatment condition. (G-I) ELISA quantification displaying the expression levels of inflammatory factors (TNF-α, IL-6, IL-1β) following LPS treatment in TGFBI knockdown/overexpression conditions. *P<0.05.

Subsequently, we investigated whether TGFBI exerts effects on other aspects of cellular function. In response to LPS treatment, our study observed significant effects on NP cells, particularly in terms of ECM degradation and inflammatory responses. Treatment with LPS led to a decrease in the levels of type II collagen [35] and aggrecan [36], key components of the ECM, as determined by both qRT-PCR and WB analysis. Concurrently, LPS induced an increase in the expression of matrix metalloproteinases 3 and matrix metalloproteinases 9 (MMP3 and MMP9), which are enzymes known to facilitate ECM breakdown [37] (Fig 4A–4F). Moreover, ELISA analysis revealed that LPS treatment also elevated the expression levels of inflammatory cytokines, indicating a pro-inflammatory effect (Fig 4G–4I). We examined the effect of TGFBI knockdown and overexpression on ECM degradation markers (collagen II, aggrecan, MMP3, MMP9) after LPS treatment using qRT-PCR and WB analysis. Specifically, TGFBI knockdown mitigated the LPS-induced decrease in type II collagen and aggrecan, as well as the increase in MMP3 and MMP9 expression. Conversely, TGFBI overexpression exacerbated these LPS-induced changes (Fig 4A–4F). Similarly, the pro-inflammatory effect of LPS, as indicated by increased inflammatory cytokines, was dampened by TGFBI knockdown but intensified by TGFBI overexpression (Fig 4G–4I). Thus, TGFBI promoted LPS-induced inflammatory cytokine levels and extracellular matrix degradation.

### TGFBI modulates NF-κB signaling pathway-mediated inflammatory responses and ECM degradation in NP cells

**Fig 5:**
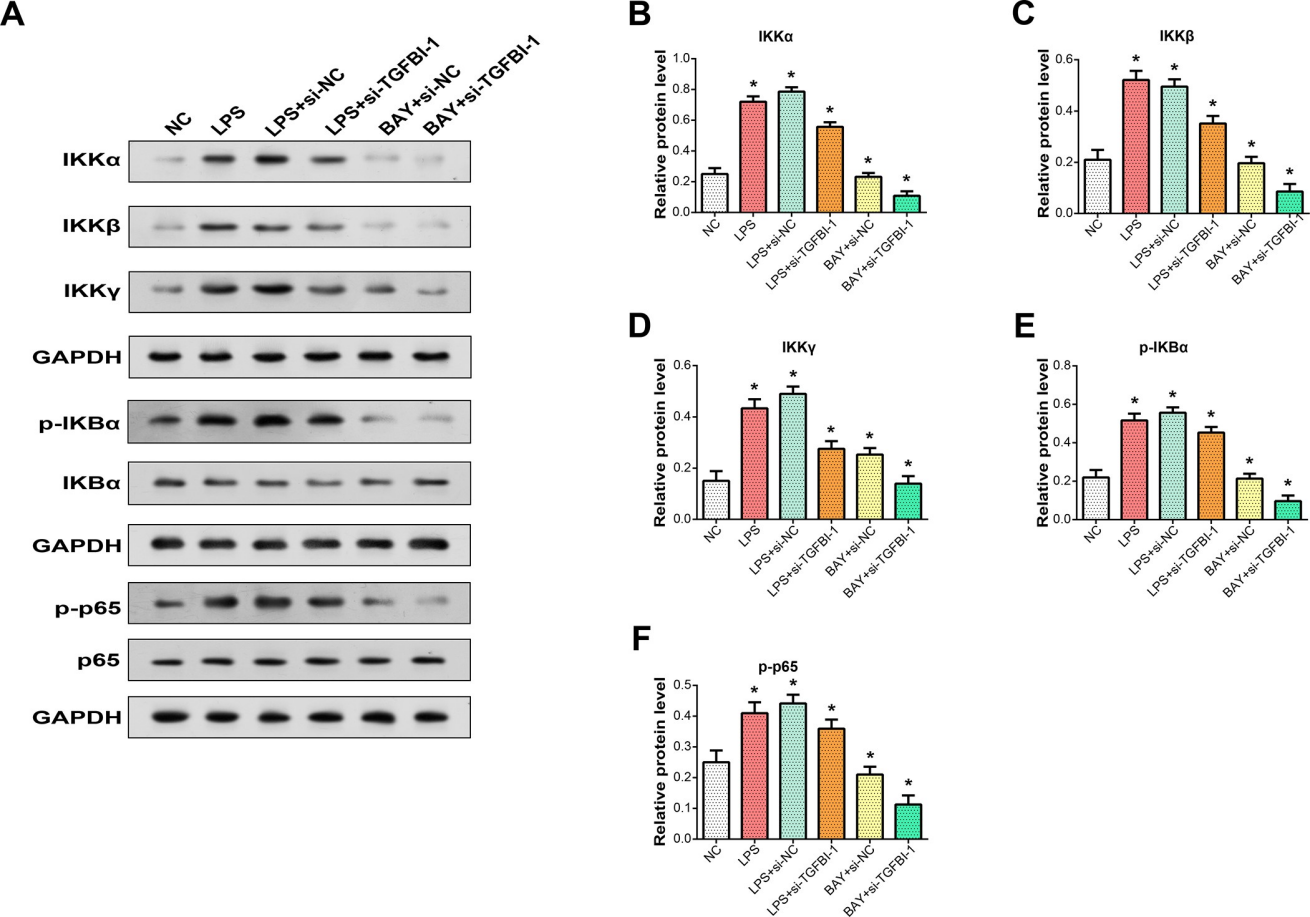
Impact of TGFBI modulation on NF-κB signaling pathway-related proteins upon LPS stimulation. (A) Western blot analysis illustrating the protein expression levels of NF-κB pathway-related proteins (IKKα, IKKβ, IKKγ, p-IKBα, IKBα, p-p65, p65) following LPS/BAY 11–7082 treatment in TGFBI knockdown/overexpression conditions. (B-F) Histograms depicting protein expression levels of NF-κB pathway-related proteins (IKKα, IKKβ, IKKγ, p-IKBα, p-p65) normalized to their respective GAPDH controls upon LPS/BAY 11–7082 stimulation in TGFBI knockdown/overexpression conditions. *P<0.05.

**Fig 6:**
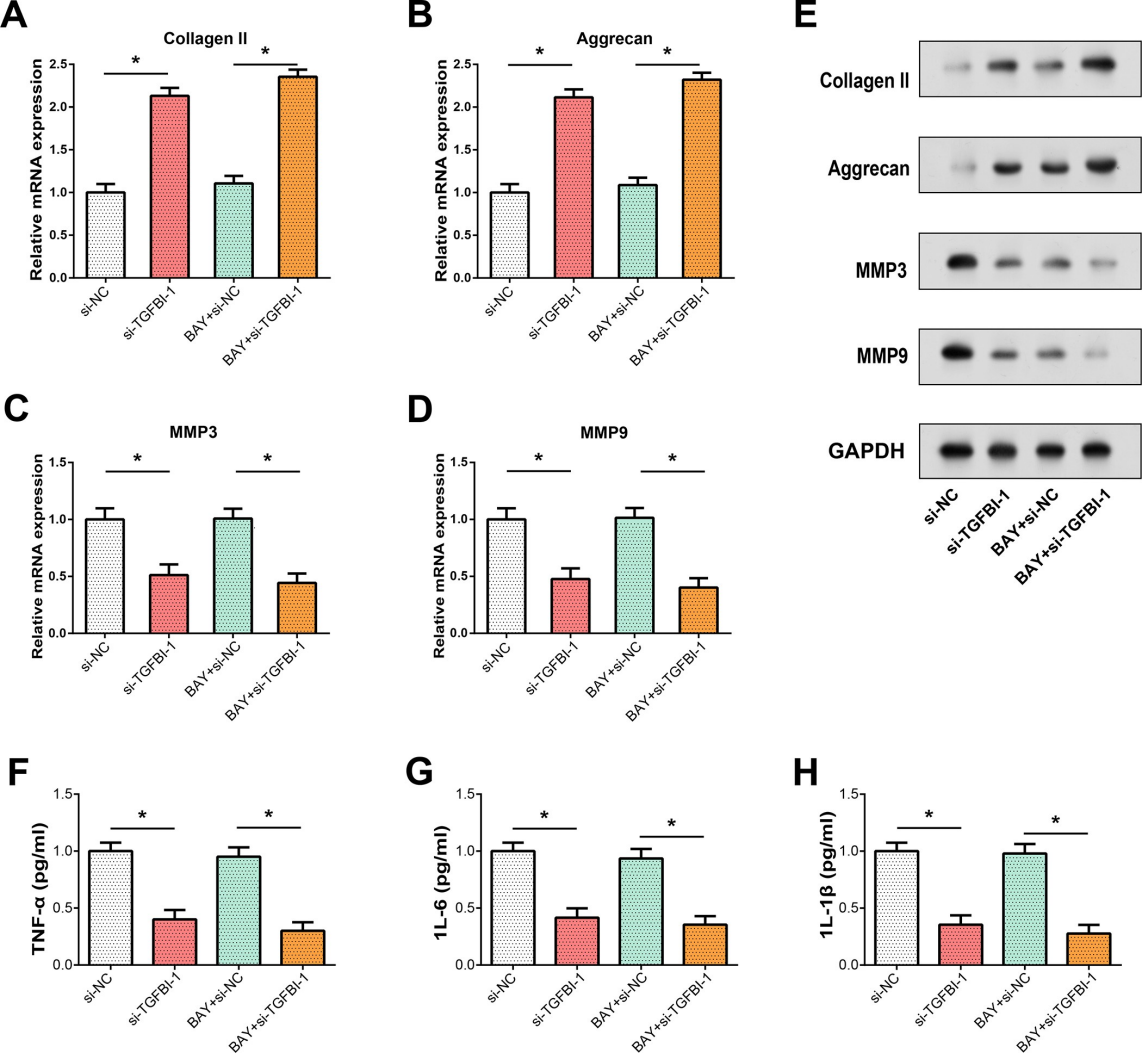
Impact of TGFBI silencing and BAY 11–7082 treatment on ECM degradation and inflammatory factors upon LPS stimulation. (A-D) PCR analysis depicting the expression levels of ECM degradation markers (Collagen II, Aggrecan, MMP3, MMP9) following LPS treatment in si-NC, si-TGFBI, BAY+si-NC, and BAY+si-TGFBI conditions. (E) Western blot analysis illustrating protein expression levels of ECM degradation markers (Collagen II, Aggrecan, MMP3, MMP9) upon LPS stimulation in si-NC, si-TGFBI, BAY+si-NC, and BAY+si-TGFBI conditions. (F-H) ELISA quantification displaying the expression levels of inflammatory factors (TNF-α, IL-6, IL-1β) upon LPS treatment in si-NC, si-TGFBI, BAY+si-NC, and BAY+si-TGFBI conditions. *P<0.05.

Given that TGFBI promotes LPS-induced cellular inflammatory responses and extracellular matrix degradation, we then explored the pathways through which it exerts these effects. We investigated the effect of TGFBI knockdown/overexpression on NF-κB pathway-related proteins (IKKα, IKKβ, IKKγ, p-IKBα, p-p6). WB analysis revealed that LPS treatment elevated the expression of NF-κB pathway-related proteins, while TGFBI knockdown suppressed their expression levels. However, the addition of BAY 11–7082 assisted the knockdown of TGFBI (Fig 5A–5F). This finding revealed that TGFBI activates the LPS-induced NF-κB pathway in NP cells. The effect of TGFBI knockdown on ECM degradation markers after LPS treatment and the additional presence of BAY 11–7082 were examined using PCR and WB analysis. The findings demonstrated that knockdown of TGFBI boosted type II collagen and collagen levels while decreasing MMP3 and MMP9 expression levels. The addition of BAY 11–7082 further enhanced these effects (Fig 6A–6E). ELISA was used to quantify TNF-α, IL-6, and IL-1β levels after LPS exposure and TGFBI knockdown. The results showed that TGFBI knockdown significantly reduced these inflammatory cytokines. This reduction was further amplified when BAY 11–7082 was added (Fig 6F–6H). Collectively, the data confirm that TGFBI affects LPS-induced inflammatory cytokine levels and ECM degradation in NP cells through the NF-κB signaling pathway.

### MARCHF8-mediated ubiquitination regulates TGFBI expression

**Fig 7:**
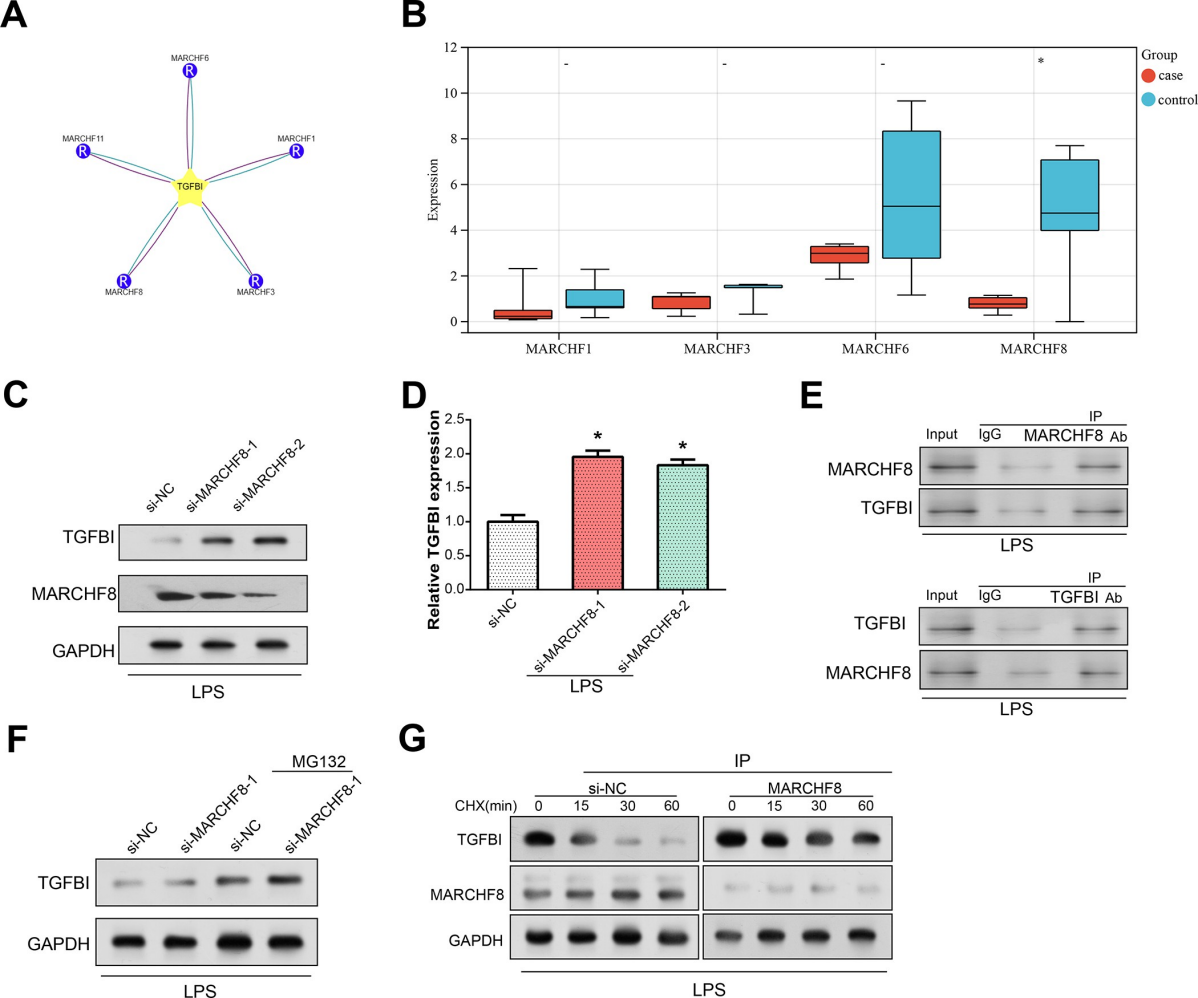
TGFBI acts as an E3 ubiquitin ligase for MARCHF8 in NP. (A) TGFBI predicted in Ubibrowser 2.0 system as the target protein of E3 ubiquitin ligase. Red lines represent predicted interactions between TGFBI and specific E3 ubiquitin ligases, indicating potential ubiquitination relationships. The blue line indicates the support of known experimental evidence for an interaction between TGFBI and the E3 ubiquitin ligase. (B) Line box plot of the expression levels of MARCHF1, MARCHF3, MARCHF6, and MARCHF8 in the GSE146904 dataset case samples and normal samples. (C) WB analysis illustrating protein expression levels of MARCHF8 and TGFBI in NP cells following LPS treatment and MARCHF8 knockdown (si-MARCHF8-1 and si-MARCHF8-2). (D) qPCR quantification showing the relative expression levels of TGFBI in NP cells following LPS treatment and MARCHF8 knockdown (si-MARCHF8-1 and si-MARCHF8-2). (E) Co-IP detection of potential interactions between MARCHF8 and TGFBI in NP cells under LPS-treated conditions. Input (total cell lysate), IgG (negative control immunoglobulin), MARCHF8 (target protein), and Antibody (specific anti-TGFBI antibody) represent different components for immunoprecipitation. (F) WB analysis demonstrating changes in TGFBI protein expression in NP cells with MARCHF8 knockdown upon treatment with the proteasome inhibitor MG132. (G) WB analysis assessing the expression of TGFBI and MARCHF8 in si-NC and si-MARCHF8 knockdown NP cells treated with the protein synthesis inhibitor CHX at various time points (0 min, 15 min, 30 min, 60 min). *P<0.05.

To explore the upstream or downstream proteins involved in the action of TGFBI, we conducted an analysis using Ubibrowser 2.0 and identified TGFBI as a potential substrate for E3 ubiquitin ligases, including MARCHF1, MARCHF3, MARCHF6, MARCHF8, and MARCHF11. Ubiquitin as a small protein modifier that plays a role in various cellular processes, including targeting proteins for degradation. The GSE146904 dataset revealed significant differences in MARCHF8 expression levels between case and normal samples, prompting the selection of MARCHF8 for follow-up studies (Fig 7A and 7B). Through WB and quantitative real-time PCR analysis, a significant increase in the expression levels of both MARCHF8 and TGFBI following MARCHF8-1 knockdown and MARCHF8-2 knockdown (Fig 7C and 7D). Co-immunoprecipitation (Co-IP) assays highlighted a potential interaction between MARCHF8 and TGFBI in LPS-treated NP cells (Fig 7E). Furthermore, WB analysis showed that TGFBI protein levels were increased after the addition of proteasome inhibitors MG132 and MARCHF8 knockdown (Fig 7F). Notably, studies of TGFBI expression in cells exposed to LPS, MARCHF8 knockdown, and the protein synthesis inhibitor cycloheximide (CHX) demonstrated increased TGFBI protein expression (Fig 7G). The time points at 0, 15, 30, and 60 minutes post-CHX treatment were selected to monitor the early degradation dynamics of TGFBI protein, as these intervals correspond to the typical protein half-life and allow observation of rapid changes in expression levels. This approach provides insights into the protein’s stability, capturing both immediate and potential delayed effects on TGFBI levels. Collectively, these findings strengthen the concept of a MARCHF8-TGFBI interaction, in which MARCHF8 regulates TGFBI expression through a ubiquitination mechanism dependent on E3 ligase activity.

### MARCHF8-mediated ubiquitination regulates NF-κB-dependent inflammatory responses and ECM degradation through TGFBI

**Fig 8:**
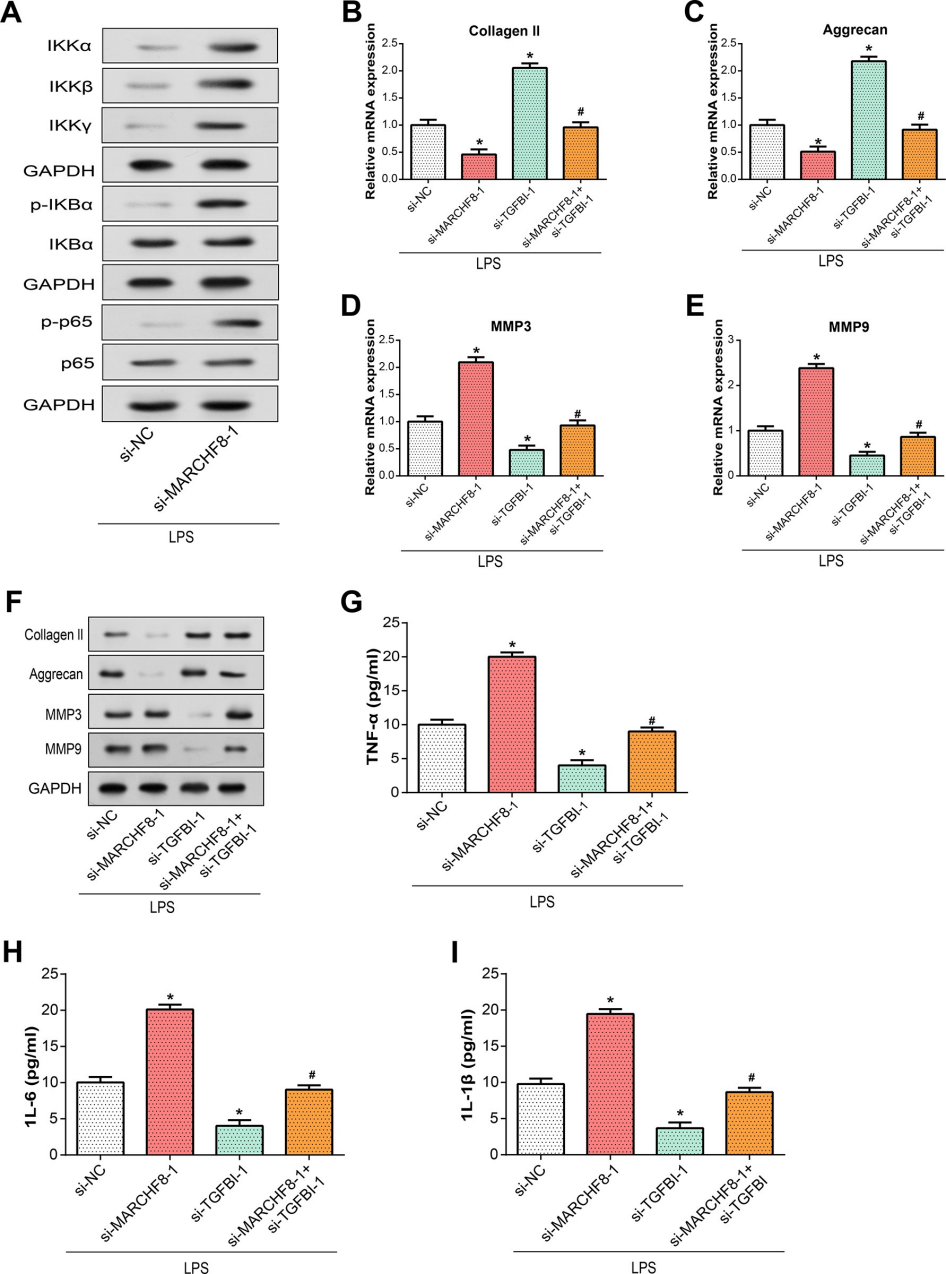
Modulation of TGFBI and MARCHF8 alters NF-κB pathways and ECM degradation markers following LPS stimulation. (A) WB analysis depicting the protein expression levels of NF-κB pathway components IKKα, IKKβ, IKKγ, p-IKBα, IKBα, p-p65, p65) upon LPS treatment in si-NC, si-TGFBI, si-MARCHF8, and si-MARCHF8+si-TGFB conditions. (B-E) qRT-PCR quantification of ECM degradation markers (Collagen II, Aggrecan, MMP3, MMP9) expression levels following LPS treatment in si-NC, si-TGFBI, si-MARCHF8, and si-MARCHF8+si-TGFB conditions. (F) WB analysis illustrating the protein expression levels of ECM degradation markers (Collagen II, Aggrecan, MMP3, MMP9) upon LPS treatment in si-NC, si-TGFBI, si-MARCHF8, and si-MARCHF8+si-TGFB conditions. (G-I) ELISA measurements displaying the expression levels of inflammatory factors (TNF-α, IL-6, IL-1β) following LPS treatment in si-NC, si-TGFBI, si-MARCHF8, and si-MARCHF8+si-TGFB conditions. *P<0.05.

Ultimately, our research discovered that MARCHF8-mediated ubiquitination of TGFBI can suppress inflammatory responses and ECM degradation by modulating the NF-κB pathway. WB analysis revealed that after MARCHF8 knockdown and LPS treatment, the protein expression level of the NF-κB signaling pathway increased (Fig 8A). PCR analysis showed that LPS treatment significantly reduced the expression levels of collagen 11 and aggrecan after MARCHF8 knockdown, but this effect was counteracted by TGFBI knockdown (Fig 8B and 8C). MARCHF8 knockdown increased the expression of MMP3 and MMP9, while TGFBI knockdown attenuated the effect of MARCHF8 knockdown (Fig 8D and 8E). ELISA detection demonstrated that MARCHF8 knockdown increased the expression of inflammatory factors, whereas TGFBI knockdown decreased their expression. Additionally, MARCHF8 knockdown hindered the impact of TGFBI knockdown (Fig 8H and 8I). These findings conclusively reveal that MARCHF8-mediated ubiquitylation of TGFBI suppresses LPS-induced inflammatory cytokine levels and extracellular matrix degradation in NP cells, possibly due to its dependence on NF-κB.

## Discussion

IDD is a multifaceted degenerative disc disease whose main symptoms include pain, limited motion, muscle weakness, numbness, altered sensation, disc herniation, spinal stenosis, and osteoarthritis, among others [38]. The diagnostic approach to IDD includes clinical evaluation, imaging techniques such as magnetic resonance imaging (MRI), and discography [39,40]. MRI can show discs dehydration, herniation, and structural abnormalities. A discogram involves injecting a contrast agent into the discs to assess its integrity and determine the source of pain [41]. Early diagnosis can help prevent or slow the progression of IDD and provide patients with more treatment options [12]. The epidemiological landscape reveals the widespread prevalence of IDD in diverse populations, highlighting the urgency to address its impact [2]. Currently, there is no definitive cure for IDD, and the prognosis for IDD varies widely, depending on factors such as severity of relapse, patient age, and comorbidities. This also indicates that the development of new diagnostic markers, therapeutic approaches, and prognostic markers is crucial to improving the treatment outcomes, quality of life, and prognosis of IDD patients.

In this study, we used the WGCNA program to create a gene co-expression network for GSE146904 and found that MEturquoise was substantially linked to IDD samples. Further DEGs analysis yielded 145 shared gene. Subsequent network analysis identified 14 potential hub genes and studied their expression in different datasets, identifying TGFBI as a hub gene. Published literature points to 24 hours as a critical time point for assessing LPS-induced inflammatory cytokine expression. Immediately afterward, we examined the expression of TGFBI in NP cells at different time points after LPS treatment. The experimental results showed that the expression of TGFBI peaked after 24 h of LPS treatment, a result consistent with published literature [42]. Therefore, we chose to collect samples for analysis at 24 h after LPS treatment in our subsequent experiments, and this time point not only reflected the significant changes in TGFBI expression, but also coincided with the findings in the existing literature on the effects of LPS on NP cell behavior.

To further elucidate the regulatory role of LPS on TGFBI expression, a series of transfection experiments were performed on NP cells in this study. These experiments included knockdown of TGFBI using siRNA and overexpression of TGFBI by transfection of expression vectors. qPCR and WB assays were used to assess the expression level of TGFBI. The results showed that LPS treatment significantly up-regulated TGFBI expression at both mRNA and protein levels. These findings suggest that LPS can activate the transcription and translation of the TGFBI gene through specific signaling pathways, thereby affecting cellular functions. And LPS treatment increases the apoptosis rate of NP cells by upregulating TGFBI expression. In the study of apoptosis, the levels and activities of these proteins are often used to assess the apoptotic state of cells. the ratio of Bax to Bcl-2 can reflect the balance of cell survival and death signaling, and the presence of Cleaved caspase-3 and Cleaved caspase-9 indicates that the apoptotic pathway has been activated [43–45]. In the experiment, the expression level of these proteins can be detected by Western Blot and other techniques to understand the activation of apoptosis signaling pathway. Our findings are consistent with the published literature that LPS treatment up-regulates the expression of Bax, Cleaved caspase-3 and Cleaved caspase-9 and down-regulates the expression of Bcl-2.

ECM refers to the noncellular components of tissues and consists of various proteins, glycoproteins, proteoglycans, and other macromolecules [46]. In IDD, the imbalance between the degradation and synthesis of ECM components in NP cells is an important reason for its occurrence [47]. ECM components such as collagen and proteoglycans maintain disc structure and hydration. As it degrades, changes in ECM composition result in lower water content, impaired load-bearing capacity, and increased likelihood of damage. Degeneration-associated changes in the ECM disrupt the delicate balance between anabolic and catabolic processes. Imbalances in the ECM metabolism led to reduced proteoglycan content, altered collagen organization, and increased expression of matrix metalloproteinases (MMPs) [48]. These ECM alterations lead to the tissue inflammation, mechanical instability, and nerve compression associated with IDD [49]. Understanding changes in the ECM in IDD offers potential therapeutic targets. Strategies to restore ECM homeostasis, such as promoting ECM synthesis and inhibiting ECM degradation, may slow or even reverse disc degeneration [50]. Subsequent *in vitro* cell experiments found that TGFBI promoted LPS-induced apoptosis in NP cells. LPS treatment decreased levels of ECM degradation markers (collagen II, aggrecan) but increased levels of matrix metalloproteinases (MMP3, MMP9), an effect that was counteracted by TGFBI knockdown and exacerbated by TGFBI overexpression of this effect. And LPS enhanced the expression of inflammatory factors, which were attenuated by TGFBI knockdown and enhanced by TGFBI overexpression. Thus, TGFBI may affect the process of IDD by promoting LPS-induced inflammatory cytokine levels and extracellular matrix degradation.

The NF-κB (nuclear factor-κB) signaling pathway is an important cell signaling pathway that is widely involved in the regulation of various biological processes such as immunity, inflammation, cell survival and proliferation [51]. In the inactive state, NF-κB is bound to the IκB protein. When subjected to external stimuli, such as inflammatory factors, oxidative stress, etc., IκB protein is phosphorylated, leading to its degradation, thereby releasing NF-κB [52]. Activated NF-κB enters the nucleus, binds to the promoter region of the target gene, and regulates the transcription of related genes. In recent years, increasing evidence has shown that the NF-κB pathway plays a key role in the synthesis, degradation and remodeling of the ECM. H Mao et al. showed that activation of PPARγ to regulate NF-κB signaling can inhibit IL-1β-induced inflammation, apoptosis and extracellular matrix degradation in chondrocytes [53]. The study by X Ao et al showed that angiopoietin-2 promotes the degradation of extracellular matrix of annulus fibrosus through HIF-1α/NF-κB signaling pathway [54]. Our study showed that Our research shows that TGFBI knockdown decreased the expression of NF-κB pathway-related proteins, but LPS treatment elevated the expression of these proteins. Supplementation BAY 11–7082 further enhanced the effect of knockdown of TGFBI on markers of ECM degradation. After knocking down TGFBI, the expression levels of inflammatory factors were significantly reduced, while the addition of BAY 11–7082 enhanced this effect. Taken together, our data confirm that TGFBI affects LPS-induced inflammatory cytokine levels and ECM degradation in NP cells through the NF-κB signaling pathway.

Subsequent bioinformatics analysis and *in vitro* cell experiments found that MARCHF8-mediated ubiquitination regulates TGFBI expression. An E3 ubiquitin ligase called MARCHF8 links ubiquitin to substrate proteins to encourage their degradation, which is essential for the control of cell membrane proteins. MARCHF8 guides the target protein into the cytoplasmic system by adding ubiquitin molecules, thereby degrading or regulating its stability and function. This process has an important impact on biological processes such as cell signal transduction, immune regulation, and cell membrane protein traffic. Knockdown of MARCHF8 led to increased expression of NF-κB signaling pathway proteins after LPS treatment. MARCHF8 knockdown affected the expression of ECM degradation markers, whereas TGFBI knockdown antagonized the effect of MARCHF8 knockdown. And MARCHF8 knockdown led to up-regulation of inflammatory cytokine expression, while MARCHF8 knockdown had an antagonistic effect on the inhibitory effect of TGFBI knockdown. These findings highlight the critical function of NF-κB signaling pathway-dependent MARCHF8-mediated TGFBI ubiquitination in the control of LPS-induced inflammatory cytokine levels and extracellular matrix breakdown in NP cells.

**Fig 9:**
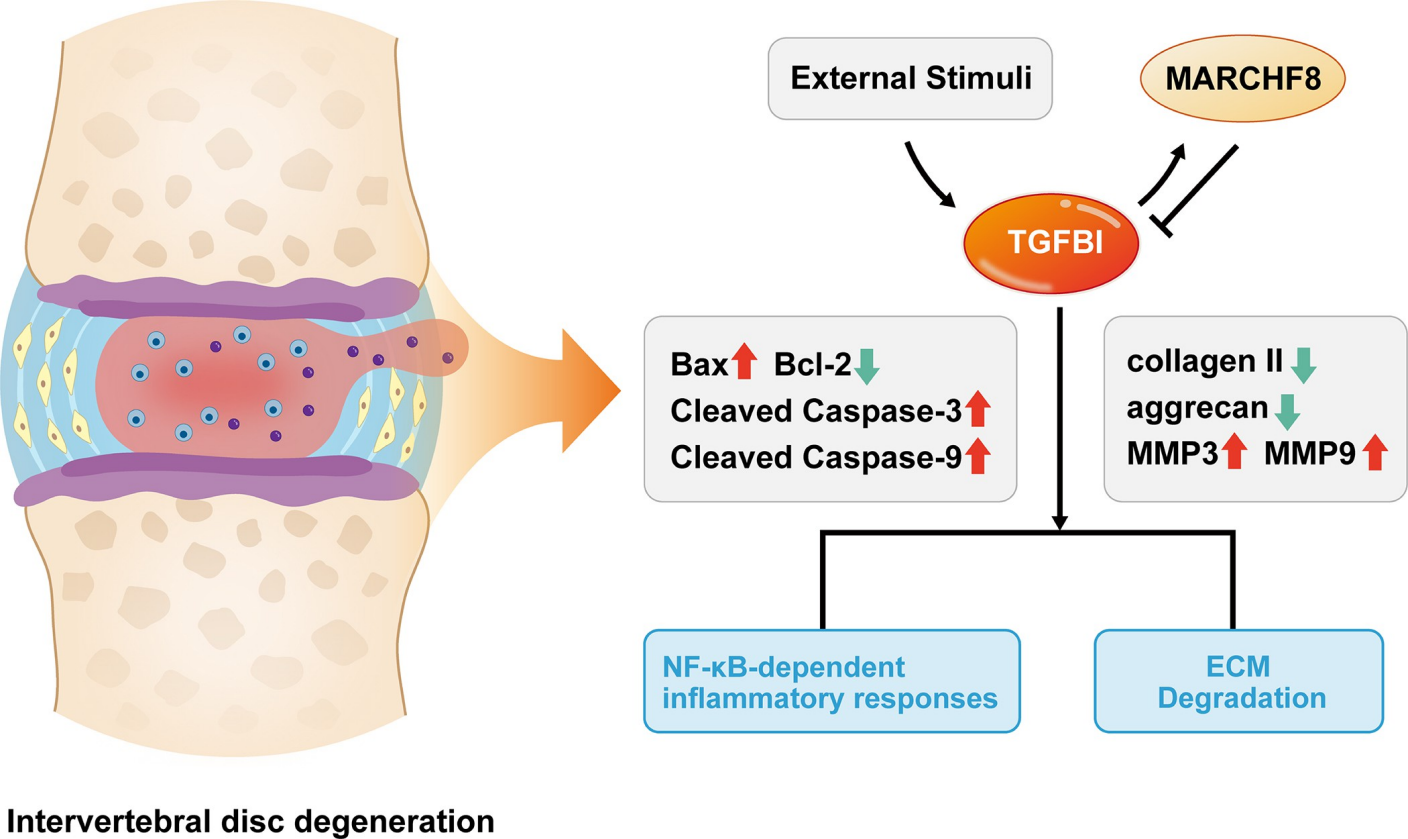
Map of the regulatory relationship between MARCHF8, TGFBI and NF-κB in disc degeneration. MARCHF8 inhibits the expression of TGFBI through a ubiquitination mechanism, whereas TGFBI plays an important role in IDD by activating the NF-κB signalling pathway, which promotes ECM degradation and inflammatory responses, as well as influencing the apoptotic process of NP cells.

In conclusion, our study reveals a complex interplay between MARCHF8, TGFBI, and NF-κB signaling pathway, and their combined effects on NP cell inflammatory response and ECM degradation during IDD (Fig 9). We discovered that LPS-induced stimulation of the NF-B signaling pathway is controlled by the MARCHF8-mediated ubiquitination, which functions as a regulating mechanism of TGFBI production. Knockdown of TGFBI counteracted the effect of the MARCHF8 knockdown, emphasizing the interconnected nature of these regulatory elements. The coordinated response of type II collagen, aggrecan, MMP3, and MMP9 to TGFBI regulation highlights its critical role in ECM dynamics. Furthermore, the effect of TGFBI on the apoptotic capacity of NP cells further enhances its multiple roles in the development of IDD. Together, all of our research points to a critical role for the MARCHF8-TGFBI axis in the control of NF-κB signaling pathway-driven inflammatory responses and ECM breakdown in NP cells. These insights highlight potential therapeutic avenues targeting this axis in an effort to alleviate the progression of IDD and related symptoms.

While our study provides compelling evidence for the role of TGFBI and MARCHF8 in IDD, it is not without limitations. The in vitro nature of our experiments necessitates further validation in in vivo models to fully understand the systemic effects and potential side effects of modulating these proteins. Future studies should also explore the upstream and downstream effectors of the MARCHF8-TGFBI axis, as well as their interaction with other signaling pathways that may influence IDD pathogenesis.

## Supporting information

S1 Graphical abstract MARCHF8 regulates TGFBI in disc degeneration via NF-κB and ubiquitination mechanisms. By analyzing the GSE146904 dataset, we applied weighted gene co-expression network analysis(WGCNA) to reveal the MEturquoise module, which is closely associated with disc degeneration. Subsequent integrative analyses revealed TGFBI, a key gene associated with disc degeneration. in vitro findings revealed a deterministic role of MARCHF8 on the expression of TGFBI,which promotes apoptosis of the nucleus pulposus (NPS)-stimulated nucleus pulposus (NPC) and destruction of the extracellular matrix(ECM). Regulation of TGFBI levels affects these effects and the NF-κB signaling pathway, thereby influencing the concentration of inflammatory cytokines. Moreover, MARCHF8 regulates TGFBI expression through ubiquitination, further controlling NP cell apoptosis, ECM degradation, and inflammatory responses, thus significantly influencing the progression of disc degeneration. This comprehensive study provides important insights into the molecular mechanisms of disc degeneration and highlights potential therapeutic targets.
(DOCX)

S1 Table Comparison of specific values of 14 overlapping genes between case and control samples in GSE146904 and GSE34094. This table summarizes the *p*-values and log_2_ fold changes of 14 overlapping genes between case and control samples in the GSE146904 and GSE34094 datasets. In GSE146904, gene expression levels are measured in RPKM, providing raw expression data that reflects the absolute abundance of transcripts. In contrast, the GSE34094 dataset presents log_2_-transformed normalized expression values, representing processed and scaled gene expression levels. By comparing these datasets, differences in gene expression trends can be observed, with statistical significance indicated by *p*-values and the magnitude of differential expression represented as log_2_ fold changes. This dual-dataset approach offers complementary insights into the gene expression profiles of degenerative disc cases and control samples.
(XLSX)

S1 Raw images Raw images of western blot analysis of key molecular markers. (PDF)

## References

[1] Xiang Q, Zhao Y, Lin J, Jiang S, Li W. The Nrf2 antioxidant defense system in intervertebral disc degeneration: Molecular insights. Experimental & Molecular Medicine. 2022;54(8):1067–75. doi: 10.1038/s12276-022-00829-6 35978054 PMC9440120

[2] Oichi T, Taniguchi Y, Oshima Y, Tanaka S, Saito T. Pathomechanism of intervertebral disc degeneration. JOR spine. 2020;3(1):e1076. doi: 10.1002/jsp2.1076 32211588 PMC7084053

[3] Kirnaz S, Capadona C, Wong T, Goldberg JL, Medary B, Sommer F, et al. Fundamentals of intervertebral disc degeneration. World Neurosurgery. 2022;157:264–73. doi: 10.1016/j.wneu.2021.09.066 34929784

[4] van Uden S, Silva-Correia J, Oliveira JM, Reis RL. Current strategies for treatment of intervertebral disc degeneration: substitution and regeneration possibilities. Biomaterials research. 2017;21(1):1–19. doi: 10.1186/s40824-017-0106-6 29085662 PMC5651638

[5] Wang Y, Cheng H, Wang T, Zhang K, Zhang Y, Kang X. Oxidative stress in intervertebral disc degeneration: Molecular mechanisms, pathogenesis and treatment. Cell Proliferation. 2023:e13448. doi: 10.1111/cpr.13448 36915968 PMC10472537

[6] Kang L, Zhang H, Jia C, Zhang R, Shen C. Epigenetic Modifications of Inflammation in Intervertebral Disc Degeneration. Ageing Research Reviews. 2023:101902. doi: 10.1016/j.arr.2023.101902 36871778

[7] Patil P, Niedernhofer LJ, Robbins PD, Lee J, Sowa G, Vo N. Cellular senescence in intervertebral disc aging and degeneration. Current molecular biology reports. 2018;4:180–90. doi: 10.1007/s40610-018-0108-8 30473991 PMC6248341

[8] Costăchescu B, Niculescu A-G, Teleanu RI, Iliescu BF, Rădulescu M, Grumezescu AM, et al. Recent advances in managing spinal intervertebral discs degeneration. International Journal of Molecular Sciences. 2022;23(12):6460. doi: 10.3390/ijms23126460 35742903 PMC9223374

[9] Wocial K, Feldman BA, Mruk B, Sklinda K, Walecki J, Waśko M. Imaging features of the aging spine. Polish Journal of Radiology. 2021;86:e380. doi: 10.5114/pjr.2021.107728 34322188 PMC8297484

[10] Yang M, Wang N, Zhang W, Sun T, Zhang D, Chang Y, et al. The Dual Effect of Abnormal Serum Uric Acid on Intervertebral Disc Degeneration. Oxidative Medicine and Cellular Longevity. 2021;2021:1–9. doi: 10.1155/2021/2362799 34630846 PMC8494577

[11] Lyu F-J, Cui H, Pan H, Mc Cheung K, Cao X, Iatridis JC, et al. Painful intervertebral disc degeneration and inflammation: from laboratory evidence to clinical interventions. Bone Research. 2021;9(1):7. doi: 10.1038/s41413-020-00125-x 33514693 PMC7846842

[12] Mahyudin F, Prakoeswa CRS, Notobroto HB, Tinduh D, Ausrin R, Rantam FA, et al. An update of current therapeutic approach for Intervertebral Disc Degeneration: A review article. Annals of Medicine and Surgery. 2022;77:103619. doi: 10.1016/j.amsu.2022.103619 35638079 PMC9142636

[13] Xin J, Wang Y, Zheng Z, Wang S, Na S, Zhang S. Treatment of intervertebral disc degeneration. Orthopaedic Surgery. 2022;14(7):1271–80. doi: 10.1111/os.13254 35486489 PMC9251272

[14] Silwal P, Nguyen-Thai AM, Mohammad HA, Wang Y, Robbins PD, Lee JY, et al. Cellular Senescence in Intervertebral Disc Aging and Degeneration: Molecular Mechanisms and Potential Therapeutic Opportunities. Biomolecules. 2023;13(4):686. doi: 10.3390/biom13040686 37189433 PMC10135543

[15] Tendulkar G, Chen T, Ehnert S, Kaps H-P, Nüssler AK. Intervertebral disc nucleus repair: hype or hope? International Journal of Molecular Sciences. 2019;20(15):3622. doi: 10.3390/ijms20153622 31344903 PMC6696292

[16] Vadalà G, Ambrosio L, Russo F, Papalia R, Denaro V. Stem cells and intervertebral disc regeneration overview—what they can and can’t do. International journal of spine surgery. 2021;15(s1):40–53. doi: 10.14444/8054 34376495 PMC8092931

[17] Ni KN, Ye L, Zhang YJ, Fang JW, Yang T, Pan WZ, et al. Formononetin improves the inflammatory response and bone destruction in knee joint lesions by regulating the NF-kB and MAPK signaling pathways. Phytother Res. 2023;37(8):3363–79. doi: 10.1002/ptr.7810 37002905

[18] Xu G, Liu C, Jiang J, Liang T, Yu C, Qin Z, et al. A novel mechanism of intervertebral disc degeneration: imbalance between autophagy and apoptosis. Epigenomics. 2020;12(13):1095–108. doi: 10.2217/epi-2020-0079 32285684

[19] Bachmeier BE, Nerlich A, Mittermaier N, Weiler C, Lumenta C, Wuertz K, et al. Matrix metalloproteinase expression levels suggest distinct enzyme roles during lumbar disc herniation and degeneration. Eur Spine J. 2009;18(11):1573–86. doi: 10.1007/s00586-009-1031-8 19466462 PMC2899407

[20] Le Maitre CL, Hoyland JA, Freemont AJ. Catabolic cytokine expression in degenerate and herniated human intervertebral discs: IL-1beta and TNFalpha expression profile. Arthritis Res Ther. 2007;9(4):R77. doi: 10.1186/ar2275 17688691 PMC2206382

[21] Kim TW, Kim AG, Lee KH, Hwang MH, Choi H. Microfluidic Electroceuticals Platform for Therapeutic Strategies of Intervertebral Disc Degeneration: Effects of Electrical Stimulation on Human Nucleus Pulposus Cells under Inflammatory Conditions. Int J Mol Sci. 2022;23(17). doi: 10.3390/ijms231710122 36077518 PMC9456475

[22] Corona A, Blobe GC. The role of the extracellular matrix protein TGFBI in cancer. Cellular Signalling. 2021;84:110028. doi: 10.1016/j.cellsig.2021.110028 33940163

[23] Costanza B, Rademaker G, Tiamiou A, De Tullio P, Leenders J, Blomme A, et al. Transforming growth factor beta‐induced, an extracellular matrix interacting protein, enhances glycolysis and promotes pancreatic cancer cell migration. International journal of cancer. 2019;145(6):1570–84. doi: 10.1002/ijc.32247 30834519

[24] Costanza B, Rademaker G, Tiamiou A, De Tullio P, Leenders J, Blomme A, et al. TGFBI, an ECM interacting protein, enhances glycolysis and promotes pancreatic cancer cell migration. International Journal of Cancer. 2019. 10.1002/ijc.32247 30834519

[25] Huang H, Tang Q, Li S, Qin Y, Zhu G. TGFBI: A novel therapeutic target for cancer. Int Immunopharmacol. 2024;134:112180. doi: 10.1016/j.intimp.2024.112180 38733822

[26] Wang B-j, Chi K-p, Shen R-l, Zheng S-w, Guo Y, Li J-f, et al. TGFBI promotes tumor growth and is associated with poor prognosis in oral squamous cell carcinoma. Journal of Cancer. 2019;10(20):4902. doi: 10.7150/jca.29958 31598162 PMC6775518

[27] Puthdee N, Sriswasdi S, Pisitkun T, Ratanasirintrawoot S, Israsena N, Tangkijvanich P. The LIN28B/TGF-β/TGFBI feedback loop promotes cell migration and tumour initiation potential in cholangiocarcinoma. Cancer gene therapy. 2022;29(5):445–55. 34548635 10.1038/s41417-021-00387-5 PMC9113936

[28] Chao-Shern C, DeDionisio LA, Jang J-H, Chan CC, Thompson V, Christie K, et al. Evaluation of TGFBI corneal dystrophy and molecular diagnostic testing. Eye. 2019;33(6):874–81. doi: 10.1038/s41433-019-0346-x 30760895 PMC6707296

[29] Kheir V, Cortés‐González V, Zenteno JC, Schorderet DF. Mutation update: TGFBI pathogenic and likely pathogenic variants in corneal dystrophies. Human mutation. 2019;40(6):675–93. doi: 10.1002/humu.23737 30830990

[30] Chen Y, Zhao H, Feng Y, Ye Q, Hu J, Guo Y, et al. Pan-cancer analysis of the associations of TGFBI expression with prognosis and immune characteristics. Frontiers in Molecular Biosciences. 2021;8:745649. doi: 10.3389/fmolb.2021.745649 34671645 PMC8521171

[31] Dowling P, Gargan S, Zweyer M, Swandulla D, Ohlendieck K. Extracellular Matrix Proteomics: The mdx-4cv Mouse Diaphragm as a Surrogate for Studying Myofibrosis in Dystrophinopathy. Biomolecules. 2023;13(7):1108. doi: 10.3390/biom13071108 37509144 PMC10377647

[32] Xu Y, Zhang D, Ji J, Zhang L. Ubiquitin ligase MARCH8 promotes the malignant progression of hepatocellular carcinoma through PTEN ubiquitination and degradation. Mol Carcinog. 2023;62(7):1062–72. doi: 10.1002/mc.23546 37098835

[33] Yang X, Shi C, Li H, Shen S, Su C, Yin H. MARCH8 attenuates cGAS-mediated innate immune responses through ubiquitylation. Sci Signal. 2022;15(732):eabk3067. doi: 10.1126/scisignal.abk3067 35503863

[34] Sun D, Kong N, Dong S, Chen X, Qin W, Wang H, et al. 2AB protein of Senecavirus A antagonizes selective autophagy and type I interferon production by degrading LC3 and MARCHF8. Autophagy. 2022;18(8):1969–81. doi: 10.1080/15548627.2021.2015740 34964697 PMC9450971

[35] Eyre DR. Collagens of the disc. Biology Of Invertebral Disc: CRC press; 2019. P. 171–88.

[36] Bertrand J, Held A. Role of proteoglycans in osteoarthritis. Cartilage: Volume 2: Pathophysiology. 2017:63–80.

[37] Luchian I, Goriuc A, Sandu D, Covasa M. The role of matrix metalloproteinases (MMP-8, MMP-9, MMP-13) in periodontal and peri-implant pathological processes. International Journal of Molecular Sciences. 2022;23(3):1806. doi: 10.3390/ijms23031806 35163727 PMC8837018

[38] Khan AN, Jacobsen HE, Khan J, Filippi CG, Levine M, Lehman RA, et al. Inflammatory biomarkers of low back pain and disc degeneration: a review. Annals of the new ork academy of sciences. 2017;1410(1):68–84. doi: 10.1111/nyas.13551 29265416 PMC5744892

[39] Wang W, Hou J, Lv D, Liang W, Jiang X, Han H, et al. Multimodal quantitative magnetic resonance imaging for lumbar intervertebral disc degeneration. Experimental and Therapeutic Medicine. 2017;14(3):2078–84. doi: 10.3892/etm.2017.4786 28962127 PMC5609182

[40] Wang Z-X, Hu Y-G. Imaging analysis of the high-intensity zone on lumbar spine magnetic resonance images: classification, features and correlation with low back pain. Journal of Pain Research. 2021:2981–9. doi: 10.2147/JPR.S332509 34588808 PMC8473715

[41] Calodney A, Vest AT. Discography. Regenerative Medicine: A Complete Guide for Musculoskeletal and Spine Disorders: Springer; 2022. P. 155–81.

[42] Saban MR, Hellmich H, Nguyen NB, Winston J, Hammond TG, Saban R. Time course of LPS-induced gene expression in a mouse model of genitourinary inflammation. Physiol Genomics. 2001;5(3):147–60. doi: 10.1152/physiolgenomics.2001.5.3.147 11285368

[43] Oltvai ZN, Milliman CL, Korsmeyer SJ. Bcl-2 heterodimerizes in vivo with a conserved homolog, Bax, that accelerates programmed cell death. Cell. 1993;74(4):609–19. doi: 10.1016/0092-8674(93)90509-o 8358790

[44] Li P, Nijhawan D, Budihardjo I, Srinivasula SM, Ahmad M, Alnemri ES, et al. Cytochrome c and dATP-dependent formation of Apaf-1/caspase-9 complex initiates an apoptotic protease cascade. Cell. 1997;91(4):479–89. doi: 10.1016/s0092-8674(00)80434-1 9390557

[45] Zou H, Li Y, Liu X, Wang X. An APAF-1.cytochrome c multimeric complex is a functional apoptosome that activates procaspase-9. J Biol Chem. 1999;274(17):11549–56. doi: 10.1074/jbc.274.17.11549 10206961

[46] Kusindarta DL, Wihadmadyatami H. The role of extracellular matrix in tissue regeneration. Tissue regeneration. 2018;65.

[47] Zhang GZ, Liu MQ, Chen HW, Wu ZL, Gao YC, Ma ZJ, et al. NF‐κB signalling pathways in nucleus pulposus cell function and intervertebral disc degeneration. Cell proliferation. 2021;54(7):e13057. 34028920 10.1111/cpr.13057 PMC8249791

[48] Liu J, Khalil RA. Matrix metalloproteinase inhibitors as investigational and therapeutic tools in unrestrained tissue remodeling and pathological disorders. Progress in molecular biology and translational science. 2017;148:355–420. doi: 10.1016/bs.pmbts.2017.04.003 28662828 PMC5548434

[49] Kirnaz S, Capadona C, Lintz M, Kim B, Yerden R, Goldberg JL, et al. Pathomechanism and biomechanics of degenerative disc disease: features of healthy and degenerated discs. International Journal of Spine Surgery. 2021;15(s1):10–25. doi: 10.14444/8052 34376493 PMC8092938

[50] Chen S, Liu S, Ma K, Zhao L, Lin H, Shao Z. TGF-β signaling in intervertebral disc health and disease. Osteoarthritis and Cartilage. 2019;27(8):1109–17. 31132405 10.1016/j.joca.2019.05.005

[51] Liu T, Zhang L, Joo D, Sun S-C. NF-κB signaling in inflammation. Signal transduction and targeted therapy. 2017;2(1):1–9. 10.1038/sigtrans.2017.23 PMC5661633 29158945

[52] Singh S, Singh TG. Role of nuclear factor kappa B (NF-κB) signalling in neurodegenerative diseases: an mechanistic approach. Current Neuropharmacology. 2020;18(10):918–35. 32031074 10.2174/1570159X18666200207120949 PMC7709146

[53] Mao H, Han B, Li H, Tao Y, Wu W. FABP4 knockdown suppresses inflammation, apoptosis and extracellular matrix degradation in IL-1β-induced chondrocytes by activating PPARγ to regulate the NF-κB signaling pathway. Molecular Medicine Reports. 2021;24(6):1–10. 10.3892/mmr.2021.12495 PMC8532115 34651666

[54] Ao X, Li Y, Jiang T, Li C, Lian Z, Wang L, et al. Angiopoietin‐2 Promotes Mechanical Stress‐induced Extracellular Matrix Degradation in Annulus Fibrosus Via the HIF‐1α/NF‐κB Signaling Pathway. Orthopaedic Surgery. 2023;15(9):2410–22. 37475697 10.1111/os.13797 PMC10475680

