# Supplementary material for: MARCHF8-mediated ubiquitination via TGFBI regulates NF-κB dependent inflammatory responses and ECM degradation in intervertebral disc degeneration
Source: PLoS One. 2025 Jan 3;20(1):e0314021. doi: 10.1371/journal.pone.0314021 (PMC11698339; doi:10.1371/journal.pone.0314021)
Supplement: S1 Graphical abstract — By analyzing the GSE146904 dataset, we applied weighted gene co-expression network analysis(WGCNA) to reveal the MEturquoise module, which is closely associated with disc degeneration. Subsequent integrative analyses revealed TGFBI, a key gene associated with disc degeneration. in vitro findings revealed a deterministic role of MARCHF8 on the expression of TGFBI,which promotes apoptosis of the nucleus pulposus (NPS)-stimulated nucleus pulposus (NPC) and destruction of the extracellular matrix(ECM). Regulation of TGFBI levels affects these effects and the NF-κB signaling pathway, thereby influencing the concentration of inflammatory cytokines. Moreover, MARCHF8 regulates TGFBI expression through ubiquitination, further controlling NP cell apoptosis, ECM degradation, and inflammatory responses, thus significantly influencing the progression of disc degeneration. This comprehensive study provides important insights into the molecular mechanisms of disc degeneration and highlights potential therapeutic targets. (DOCX) [file pone.0314021.s001.docx]

| **GSE146904** |
| --- |


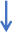


| **Common DEGs** |
| --- |


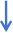


| **WGCNA analysis** |
| --- |

↓

| **Key module** |
| --- |


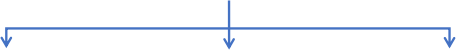
↓

| **GSE34095** |
| --- |

| **GSE46904** |
| --- |

| **PPI network** |
| --- |

| **TGFBI** |
| --- |

↓


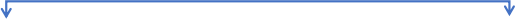


| **Cell culture**  **and**  **treatment**  **with LPS** |
| --- |

| ***In vitro***  **ubiquitination assay** |
| --- |

| **ELISA Assay** |
| --- |

| **Flow**  **Cytometry** |
| --- |

| **Co-IP Assay** |
| --- |

| **WB**  **Assay** |
| --- |

| **qRT-**  **PCR**  **Assay** |
| --- |

↓

| **MARCHF8** |
| --- |

↓


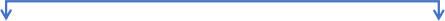


| **ECM degradation** |
| --- |

| **NF-κB-dependent**  **inflammatory responses** |
| --- |

!

| **MARCHF8-mediated ubiquitination via TGFBI regulates NF-κB**  **dependent inflammatory responses and ECM degradation in**  **intervertebral disc degeneration** |
| --- |

Performance：

By analyzing the GSE146904 dataset, we applied weighted gene co-expression network analysis (WGCNA) to reveal the MEturquoise module, which is closely associated with disc degeneration. Subsequent integrative analyses revealed TGFBI, a key gene associated with disc degeneration. in vitro findings revealed a deterministic role of MARCHF8 on the expression of TGFBI, which promotes apoptosis of the nucleus pulposus (NPS)-stimulated nucleus pulposus (NPC) and destruction of the extracellular matrix (ECM). Regulation of TGFBI levels affects these effects and the NF-κB signaling pathway, thereby influencing the concentration of inflammatory cytokines. Moreover, MARCHF8 regulates TGFBI expression through ubiquitination, further controlling NP cell apoptosis, ECM degradation, and inflammatory responses, thus significantly influencing the progression of disc degeneration. This comprehensive study provides important insights into the molecular mechanisms of disc degeneration and highlights potential therapeutic targets.
