# Supplementary material for: MARCHF8-mediated ubiquitination via TGFBI regulates NF-κB dependent inflammatory responses and ECM degradation in intervertebral disc degeneration
Source: PLoS One. 2025 Jan 3;20(1):e0314021. doi: 10.1371/journal.pone.0314021 (PMC11698339; doi:10.1371/journal.pone.0314021)
Supplement: S1 Raw images — (PDF) [file pone.0314021.s003.pdf]

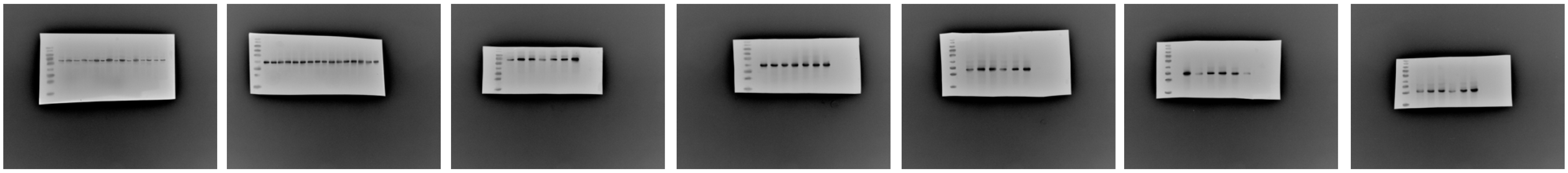

Figure 2G_TGFBI_68 kDa
Figure 2G_GAPDH_36 kDa
Figure 3B_TGFBI_68 kDa
Figure 3B_GAPDH_36 kDa
Figure 3F_Bax_ 21 kDa
Figure 3F_Bcl-2_ 26 kDa
Figure 3F_Cleaved caspase-3_17kDa

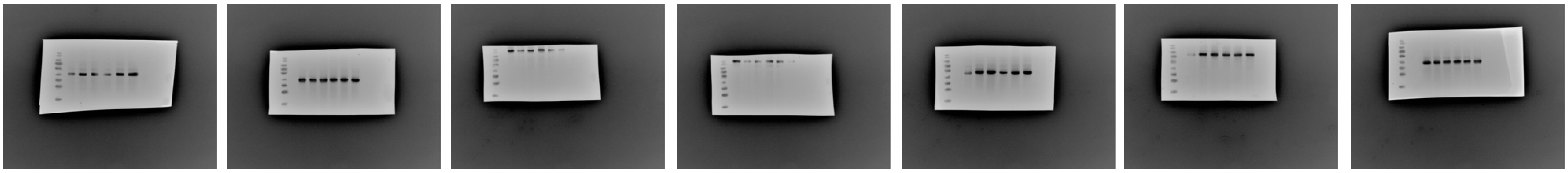

Figure 3F_Cleaved caspase-9_37kDa
Figure 3F_GAPDH_36 kDa
Figure 4E_Collagen II_142 kDa
Figure 4E_Aggrecan_250kDa
Figure 4E_MMP3_50 kDa
Figure 4E_MMP9_89 kDa
Figure 4E_GAPDH_36 kDa

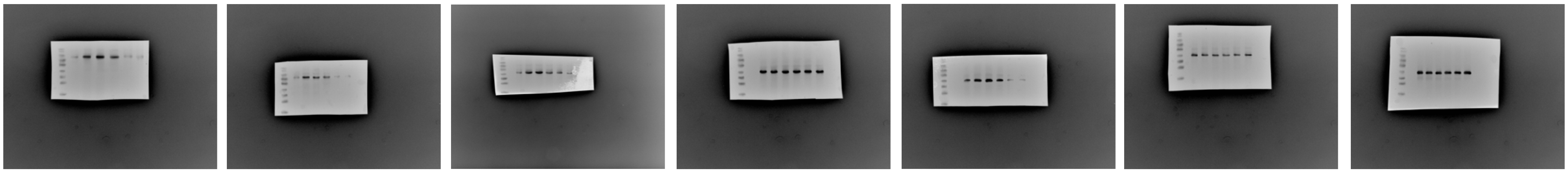

Figure 5A_IKKα_88kDa
Figure 5A_IKKβ_85 kDa
Figure 5A_IKKγ_48 kDa
Figure 5A_GAPDH_36 kDa
Figure 5A_p-IKBα_35 kDa
Figure 5A_IKBα_35 kDa
Figure 5A_GAPDH_36 kDa

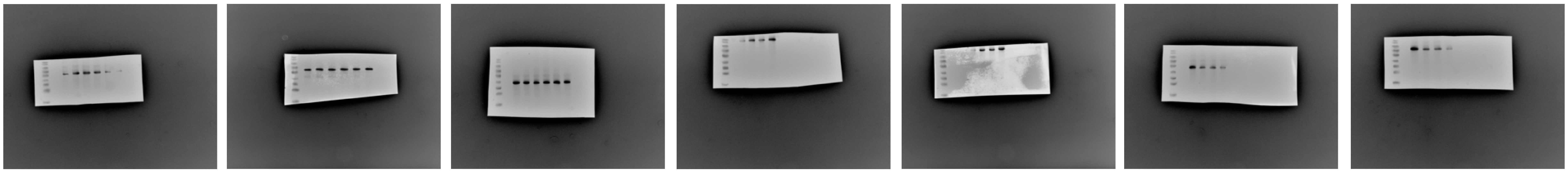

Figure 5A_p-p65_65 kDa
Figure 5A_p65_65 kDa
Figure 5A_GAPDH_36 kDa
Figure 6E_Collagen II_142 kDa
Figure 6E_Aggrecan_250kDa
Figure 6E_MMP3_50 kDa
Figure 6E_MMP9_89 kDa

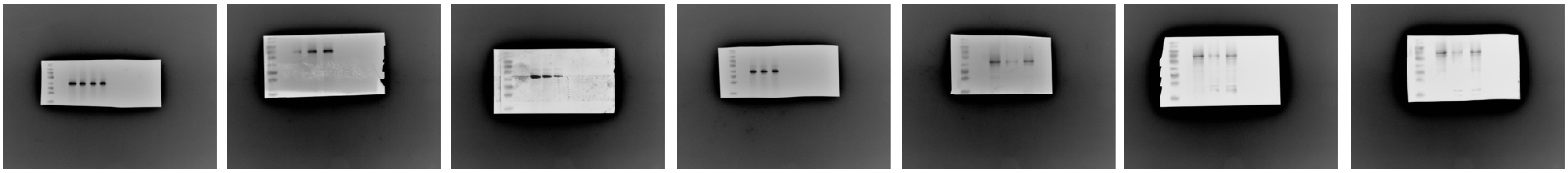

Figure 6E_GAPDH_36 kDa
Figure 7C_TGFBI_68 kDa
Figure 7C_MARCHF8_38kDa
Figure 7C_GAPDH_36 kDa
Figure 7E_MARCHF8_38kDa
Figure 7E_TGFBI_68 kDa
Figure 7E_TGFBI_68 kDa

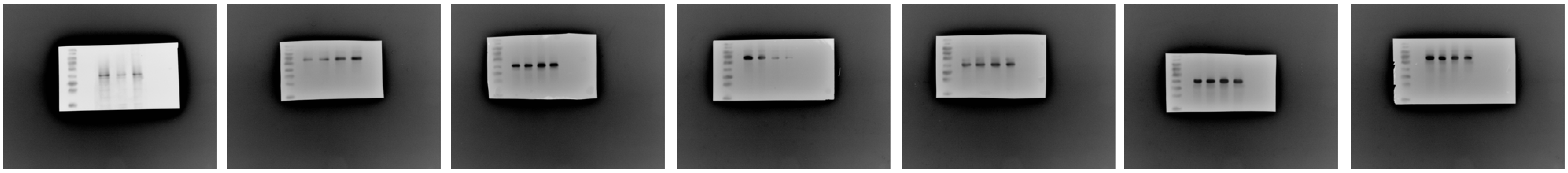

Figure 7E_MARCHF8_38kDa
Figure 7F_TGFBI_68 kDa
Figure 7F_GAPDH_36 kDa
Figure 7G_TGFBI_68 kDa
Figure 7G_MARCHF8_38kDa
Figure 7G_GAPDH_36 kDa
Figure 7G_TGFBI_68 kDa

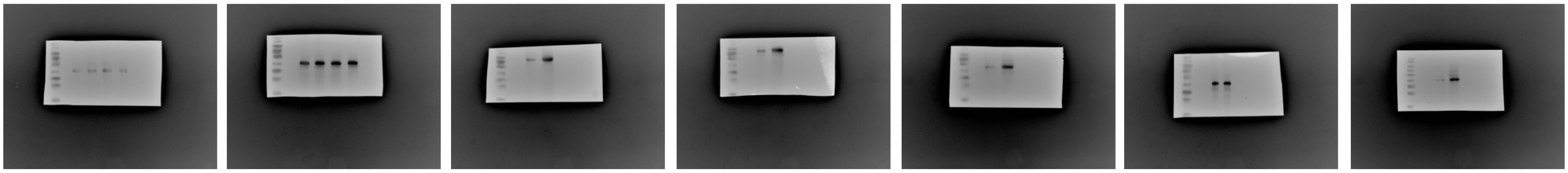

Figure 7G_MARCHF8_38kDa
Figure 7G_GAPDH_36 kDa
Figure 8A_IKKα_88kDa
Figure 8A_IKKβ_85 kDa
Figure 8A_IKKγ_48 kDa
Figure 8A_GAPDH_36 kDa
Figure 8A_p-IKBα_35 kDa

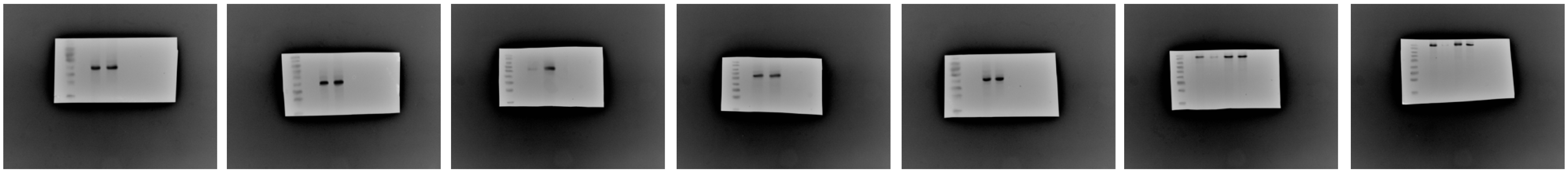

Figure 8A_IKBα_35 kDa
Figure 8A_GAPDH_36 kDa
Figure 8A_p-p65_65 kDa
Figure 8A_p65_65 kDa
Figure 8A_GAPDH_36 kDa
Figure 8F_Collagen II_142 kDa
Figure 8F_Aggrecan_250kDa

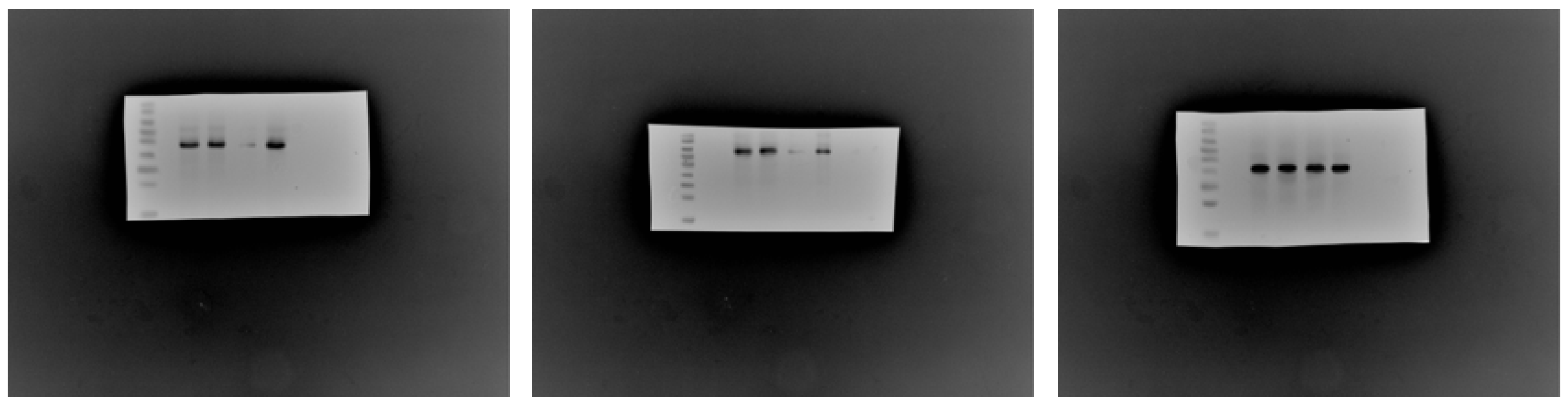

Figure 8F_MMP3_50 kDa
Figure 8F_MMP9_89 kDa
Figure 8F_GAPDH_36 kDa
